# Identifying needs in adult rehabilitation to support the clinical implementation of robotics and allied technologies: an Italian national survey

**DOI:** 10.3389/fdgth.2026.1718274

**Published:** 2026-04-01

**Authors:** Irene Giovanna Aprile, Alessio Fasano, Marco Germanotta, Maria Cristina Mauro, Monia Andrea Papa, Giovanna Nicora, Leopoldo Trieste, Giuseppe Turchetti, Elena Beani, Giuseppina Sgandurra, Silvana Quaglini, Cristina Messa, The Mission 1 Fit4MedRob Consortium

**Affiliations:** 1 IRCCS Fondazione Don Carlo Gnocchi ETS, Florence, Italy; 2 Department of Electrical, Computer and Biomedical Engineering, University of Pavia, Pavia, Italy; 3 Institute of Management, Scuola Superiore Sant’Anna, Pisa, Italy; 4 Department of Clinical and Experimental Medicine, University of Pisa, Pisa, Italy; 5 Department of Developmental Neuroscience, IRCCS Fondazione Stella Maris, Pisa, Italy; 6 IRCCS Fondazione Don Carlo Gnocchi ETS, Milan, Italy; 7 School of Medicine and Surgery, University of Milano-Bicocca, Milan, Italy

**Keywords:** healthcare services, ICF, patients' needs, rehabilitation, robotics, survey

## Abstract

**Introduction:**

Robotics and technological interventions are increasingly being explored as solutions to improve rehabilitation outcomes but their implementation in clinical practice remains very limited. Understanding patient needs is crucial for effective integration of these technologies, ensuring they align with and address the actual requirements of individuals in clinical settings. The primary aim of this study is to explore the rehabilitation needs of adults with motor, sensory, and/or cognitive disabilities in order to more effectively guide the practice of technological and robotic interventions in clinical setting.

**Methods:**

To this end, as part of the Fit for Medical Robotics Initiative, we conducted a survey targeting adult patients recruited from clinical centers participating in the Initiative. It aimed to provide a clear understanding of the patients' rehabilitation priorities, as well as perceived efficacy and satisfaction levels about the robotic and the traditional rehabilitation, in order to better address trials on the use of robots and technologies in individuals with disabilities considering a patient-centered perspective. The survey was structured on the basis of the International Classification of Functioning, Disability, and Health framework.

**Results:**

There were 424 respondents representing a range of conditions, including stroke, Parkinson's disease, multiple sclerosis, neuromuscular disorders, and other motor and cognitive impairments. Notably, 86% of respondents reported undergoing traditional rehabilitation, while 39% had experienced (also) robotic interventions, highlighting limited accessibility to advanced rehabilitation technologies. Additionally, respondents expressed a significant need for multidomain rehabilitation, with movement being the most prioritized domain. The degree of satisfaction was higher among respondents receiving technological interventions, particularly in addressing mobility. Furthermore, a substantial proportion of respondents indicated a strong need for receiving home-based care.

**Discussion:**

The patient needs identified through the survey were fundamental for designing pragmatic clinical trials, whose results will help shape the rehabilitation offer using new and innovative models.

## 1 Introduction

The integration of robots and allied digital technologies in rehabilitation has seen substantial growth in recent years, driven by the need for personalized and intensive therapeutic interventions across various patient populations (1). Despite advancements and positive clinical outcomes (2, 3), widespread adoption remains limited, primarily due to insufficient attention to patient-specific needs and barriers experienced by end-users (4–7). Understanding the diverse needs and perspectives of patients is thus essential to ensure effective and meaningful clinical implementation of rehabilitation technologies (8).

Within this context, the Italian Initiative “Fit for Medical Robotics” (Fit4MedRob)^1^ aims to bridge the existing gaps between technological innovation and clinical application in robotic rehabilitation through comprehensive research and practical implementations, integrating technological solutions across a broad spectrum of patient populations and clinical settings. Fit4MedRob is structured around distinct missions that collectively aim to facilitate clinical translation, foster technological innovation, and enhance the user-centered design of rehabilitation technologies. Mission 1 of Fit4MedRob is carrying out a set of pragmatic clinical trials with existing commercial devices that should clarify the effectiveness and sustainability of technologies for rehabilitation (9). A pivotal component for the design of these trials is the systematic identification and evaluation of patients' needs across heterogeneous clinical populations. This identification will also ensure that the non-commercial robotic solutions developed within Mission 2 align closely with end-user requirements and preferences, as well as it will guide the design of next generation of robots and technologies, devised within Mission 3.

Indeed, effective integration of robotic rehabilitation into clinical practice requires a thorough understanding of the diverse needs and expectations of patients with motor, sensory, and cognitive impairments, which reflect different clinical conditions and may differently influence the acceptance and adoption of robotics and related technologies (6, 10, 11). Patient needs can substantially differ based on the specific disorder, severity of impairments, and individual rehabilitation goals, making personalized assessments essential to ensure technologies genuinely meet user expectations and improve clinical outcomes. Additionally, it is recognized that involving patients and families in clinical decision-making processes improves health outcomes, enhances satisfaction with care, reduces healthcare costs, and benefits clinician experiences (12). Hence, methodologies capable of systematically capturing these diverse user perspectives are crucial to support broader adoption and meaningful integration into routine clinical practice (13).

Currently, there is a lack of studies providing the necessary knowledge and a clear description of patients' needs in rehabilitation, and this could be one of the barriers limiting the ability to effectively adapt and implement technological solutions in the clinical setting (9). In particular, a recent systematic review conducted by our research group highlighted significant gaps in the methodologies used to capture end-users' perspectives in robotic rehabilitation (10). The review identified that existing literature often employed semi-structured qualitative approaches with small, homogeneous samples, based on specific pathologies. There is a lack of studies capable of investigating rehabilitation needs and satisfaction with robotic rehabilitation across large and diverse patient populations. From our perspective, only by integrating standardized frameworks—such as the International Classification of Functioning, Disability, and Health (ICF) (14)—into surveys is it possible to study rehabilitation needs across large samples of individuals with disabilities. The ICF offers a comprehensive and standardized language to describe health states across multiple domains, including body functions and structures, activities, participation, and contextual factors. Leveraging the ICF framework can thus facilitate a more holistic and multidimensional understanding of patients' rehabilitation experiences, not only in terms of functional impairments but also in capturing broader aspects such as daily life integration, participation in social contexts, and personal perception of recovery. The ICF's structured and multidimensional nature enables precise identification of patient-specific requirements, improving the tailoring of rehabilitation interventions and ultimately contributing to better clinical outcomes (15). An example is the Individual Rehabilitation Project (IRP), an ICF-based rehabilitation management scheme developed with the patient and an interdisciplinary team to define functioning needs, set goals, plan interventions, and evaluate outcomes through iterative rehabilitation cycles (16). Furthermore, initiatives such as the ICF Core Set Project continue to refine and expand ICF application across various health contexts, underscoring its essential role in rehabilitation research and practice (17).

In this study, we developed and administered, within the Fit4MedRob Initiative, online surveys targeting adult patients with neurological disorders (such as stroke, Parkinson's disease, multiple sclerosis, amyotrophic lateral sclerosis, muscular dystrophies, and other neuromuscular or cognitive impairments) aimed to:

- depict the current rehabilitative scenario in Italy, including organizational and economic aspects,
- collect patients' rehabilitative needs and experiences with current care, and
- explore their opinions, expectations, and attitudes toward the use of robotics and digital technologies in rehabilitation.

In (18), we have described the methodology to develop these surveys, informed by systematic review findings (10). Here, we present the results obtained from administering these surveys.

## 2 Materials and methods

### 2.1 Surveys design

The methodology for developing the patient-centered surveys used in this study was informed by the previous systematic review on needs-assessment methodologies in the robotic rehabilitation field (10) and was comprehensively described in another paper (18). Here we briefly summarize this methodology.

Descriptive surveys were developed using the ICF framework as a foundational model, ensuring a holistic and standardized approach to capturing patients' experiences and needs. The outcome of interest was the rehabilitative care received and the rehabilitative needs of patients, with particular reference to both traditional and technological treatments, as well as all the therapeutic settings (inpatient, outpatient, home-based). With traditional rehabilitation we referred to conventional interventions delivered by trained healthcare professionals. This includes physical rehabilitation (e.g., task-oriented exercises, soft tissue and joint mobilization, strengthening, balance and mobility training, functional upper-limb tasks, and patient/caregiver education) and cognitive rehabilitation (e.g., programs targeting attention, memory, and executive functions), generally provided according to established clinical guidelines and evidence-based practice. With technological rehabilitation we referred to robotic rehabilitation, i.e., rehabilitation assisted by robots or electromechanical devices providing physical assistance or movement guidance, as well as to digital systems, including sensor-based tools, virtual reality environments, balance platforms, and other digital technologies targeting motor and/or cognitive functions. These approaches are generally implemented within structured rehabilitation programs under professional supervision. More specifically, for the purpose of the survey, we have defined and considered as robotic and allied digital technologies for rehabilitation the following eight functional categories:

- assistive (generic) and mobile servants, such as technological wheelchairs, devices for self-care or eating, eye tracking communicators and humanoid robots;
- advanced treadmills, showing enhanced functionalities such as visual feedback, motion capture system, or body weight support;
- upper limb exoskeletons, anthropometric robots that support the partial/full range of motion of the human arm;
- lower limb exoskeletons, anthropometric robots that support the partial/full range of motion of the human leg;
- lower limb end effectors, which control and support the movement of the most distal segment of the lower limb extremity;
- upper limb end effectors, which control and support the movement of the most distal segment of the upper limb extremity;
- proprioceptive/stabilometric/balance platforms, allowing for evaluation and training of balance, coordination, and proprioception;
- sensor-based devices, with or without virtual reality, permitting both physical and cognitive exercises, including systems exploiting virtual reality and exercises for improving motor and/or cognitive deficits, such as those affecting attention, memory and problem solving, without providing movement support.

The target populations of patients were the following: post-stroke, multiple sclerosis, Parkinson's disease, acquired brain injury, spinal cord injury, peripheral neuromuscular diseases, cerebral palsy (adult patients), post-oncological surgery. A further generic category, named “Others”, has been added to include possible other pathologies not attributable to the mentioned ones but sharing common characteristics with them, e.g., frail elderly, subjects affected by oncological or work-related pathologies. In particular, “work-related pathologies” referred primarily to work-related musculoskeletal disorders (WMSDs) and peripheral nerve injuries, defined as conditions affecting muscles, joints, tendons, ligaments, and nerves that are caused or aggravated by occupational activities and environments (19). WMSDs commonly include neck and low back pain, tendinitis, and carpal tunnel syndrome (20), which represent some of the most frequently reported occupational conditions. These conditions can lead to persistent pain, motor impairment, sensory disturbances (e.g., paresthesia), reduced functional capacity, and work disability, often requiring structured rehabilitation interventions (21). “Frail elderly” is an older adult, generally aged 65 years or older, characterized by reduced physiological reserve and increased vulnerability to physical and psychological stressors, who may also present comorbidities or chronic conditions that limit their ability to respond effectively to challenges (22). Recognition of frailty is essential to guide tailored rehabilitation strategies and individualized care planning, aiming to optimize functional outcomes and prevent further decline (23).

All the included subjects shared the presence of motor, sensory, and/or cognitive impairments, which may include, for example, motor impairments such as inability to walk or reduced upper-limb function; sensory impairments such as visual disturbances, altered light touch sensation, pain sensitivity, and/or altered proprioception; and cognitive impairments such as memory deficits or attention difficulties. This broad approach allowed us to consider a wide range of conditions, ensuring a comprehensive understanding of the current needs across different patient groups, and thus to gather insights that could guide the development of more inclusive and accessible robotic interventions.

No *a priori* power analysis was performed because the survey was designed primarily for descriptive needs assessment and pragmatic reach, rather than for testing a single primary hypothesis with a predefined effect size (18).

Survey development followed a structured, iterative workflow. This workflow followed the Donabedian framework (Structure–Process–Outcome). On the Structure level, a team of multidisciplinary experts (clinicians, rehabilitation engineers, and researchers) defined key domains of interest, based on existing literature. On the Process level, an iterative process was conceived in line with the COSMIN (Consensus-based Standards for the selection of health Measurement INstruments) principles to support content validity. Items were generated from literature synthesis and expert/end-user input and iteratively refined through expert review and pilot activities to improve clarity, relevance, and feasibility. Iterative consulting sessions involved stakeholder feedback from patient/caregiver associations. On the Outcome level, the survey was designed to capture priorities, perceived needs, satisfaction with current technologies, and willingness to adopt new tools, and to enable future psychometric evaluation in larger samples.

Six functional domains, common across different conditions, were identified through the mentioned workflow, matching the ICF-Core Set (24) with the specific pathologies identified as target populations in the Initiative: (1) mobility (or movement), (2) upper limb and hand use, (3) balance and postural functions, (4) self-care, (5) cognitive functions, (6) communication.

The domain *movement* refers to displacement, defined as movement for walking; walking forward, backward or sideways; walking on different surfaces, indoors or out; getting around or over obstacles; climbing up and down stairs; climbing, running, jumping. It covers mobility using specific equipment designed to facilitate movement, such as electronic or manual wheelchairs, walking frames or quadricycles, etc. The domain *upper limb function* refers to functions related to the use of the hands and arms including: perform the coordinated actions of handling objects, picking them up, manipulating them and releasing them using the hand, fingers and thumb, for example lifting coins from a table or turning a knob or picking, grasping, manipulating and releasing; perform coordinated actions necessary to move objects or manipulate them using hands and arms, for example when turning door handles or throwing or catching an object, pulling or pushing. The domain *body posture and balance* refers to functions involved in assuming, maintaining and possibly changing different postures, e.g., supine, on one's side, sitting, standing, kneeling and so on. The domain *self-care* refers to functions related to carrying out tasks related to daily routines such as preparing food and eating, pouring drinks or mixing and drinking, dressing, washing, taking care of one's body. The domain *cognitive functions* refers to cognitive and neuropsychological functions i.e., those processes involved in behaviors necessary, for instance, to find solutions to questions or situations by identifying and analyzing problems; make a choice among several options and evaluate the effects of the choice (e.g., selecting and purchasing a specific item, or deciding to undertake an activity among several ones that must be performed); perform simple or complex coordinated actions to plan, manage, and complete daily tasks, such as planning time and scheduling different activities throughout the day. The domain *communication* refers to functions related to verbal and non-verbal communication such as: understanding the literal and implicit meanings of messages in spoken language, for instance understanding whether a statement refers to a fact or is an idiomatic expression; producing words, sentences of different lengths to express a fact or tell a story orally; start, sustain and end a conversation; converse with one or more persons; use devices, techniques and other means for the purpose of communication, such as calling a friend on the phone; use telecommunication devices, technology for writing or speaking; use gestures, symbols and drawings to convey messages, such as shaking one's head to indicate disagreement or drawing a picture to convey a complex fact or idea.

Each domain incorporated a structured set of questions utilizing a 5-point Likert scale to measure aspects such as independence, impact on quality of life, satisfaction with current rehabilitation methods (including traditional and technological treatments), usability, and acceptability of robotic devices. Subjects were asked to rank all the domains in order of importance from a subjective perspective of salience. Additional open-ended questions were integrated to capture detailed qualitative insights, allowing respondents to express their experiences and specific needs more comprehensively. A final section was dedicated to economic data, i.e., asking respondents to report specific costs sustained for their rehabilitation (devices, transportation, home adaptations, special food, hired assistance and income loss for caregivers).

The survey was implemented on Microsoft Forms using conditional branching (skip logic), with questions in everyday (Italian) language. The total number of questions was 113.

Surveys were conducted anonymously, in full compliance with the General Data Protection Regulation (GDPR), and were approved by a dedicated ethics committee (joint ethics committee of the Scuola Normale Superiore and the Scuola Superiore Sant'Anna, Pisa, Italy, delibera n. 35 del 31 agosto 2023).

### 2.2 Surveys pilot testing

Prior to the main data collection, the developed survey underwent a pre-test with representative Fit4MedRob personnel (5 physicians, 8 physical therapists, and 4 biomedical engineers), as described in (18). As mentioned above, patients' and caregivers' associations were also involved to ensure clarity, comprehensibility, and relevance of the survey items. Feedback from pilot testing was used to refine wording, structure, and format of the questions to improve overall usability and respondent experience (e.g., typo corrections and rephrasing for clarity). Completion time was estimated at approximately 20–35 min, depending on the branching logic activated by respondents' answers (18).

### 2.3 Surveys administration

Since the target population included patients with various clinical conditions, multiple surveys were designed, sharing a common structural framework but differing in wording and approach to accommodate different respondent profiles: (i) a survey for adult patients capable of independently completing the questionnaire; (ii) a survey for caregivers responding on behalf of non-collaborative adult patients; (iii) a separate survey designed specifically for caregivers of pediatric patients. In this paper, we will report on anonymous surveys developed for adult patients who were able to answer the survey autonomously.

These surveys were specifically designed and administered to adult patients with above-mentioned disorders recruited through multiple clinical centers, rehabilitation centers, and patient associations across different Italian regions, ensuring a diverse and representative sample and reducing the risk of center-specific response patterns.

Surveys were distributed using an internet-based platform (Microsoft Forms), facilitating ease of access and ensuring respondents could complete them independently at their convenience. The online tool also facilitated the dissemination of the survey through weblinks via social media accounts, institutional channels of all the clinical centers involved in Fit4MedRob, and of the several patients' and caregivers' associations. In-person events (such as the Fit4MedRob conferences) were organized, to facilitate engagement.

A detailed explanatory introduction was provided at the beginning of the survey, outlining the purpose of the study, confidentiality assurances, and instructions for completion. Ethical considerations, such as informed consent, confidentiality, and anonymity, were rigorously maintained throughout the study.

The full questionnaire in its original Italian version is available at the following links: https://forms.cloud.microsoft/e/RhXbJYJEha. An English version is available as Supplementary Material of this paper.

### 2.4 Data analysis

The main features explored in the analyses were: settings of treatment (inpatient, outpatient, or home-based), type of rehabilitation (traditional vs. robotic), and the clinical context (within the National Health System or private rehabilitation service). Economic impacts, including out-of-pocket expenses and indirect costs such as loss of income, were quantified and summarized.

Survey responses were analyzed using descriptive statistics and thematic analysis methods. Quantitative data were summarized through frequency distributions, percentages, means, and standard deviations. Contingency tables were utilized to examine relationships between patient demographics (e.g., age, gender, pathology) and survey responses across the identified key functional domains. For satisfaction ratings (1–5 Likert), we used a non-parametric Mann–Whitney *U* test because the outcome is ordinal and normality cannot be assumed. Chi-squared tests were conducted to compare the proportion of respondents reporting high satisfaction (i.e., score ≥4) between traditional and robotic rehabilitation groups, with the Cramer's *V* as a measure of effect size.

Qualitative data from open-ended responses were systematically reviewed through thematic analysis to identify recurring themes, providing detailed insights into patient experiences, perceived barriers, specific rehabilitation needs and priorities, and areas requiring technological enhancements.

For data processing, anonymous survey responses were stored and analyzed using statistical software IBM SPSS Statistics for Windows, Version 29.0.1.0, as well as custom-made scripts in Python for data visualization and in-depth analysis.

Since the survey was self-administered, some items were left unanswered. For transparency, no imputation was performed; analyses used available data for each item: e.g., percentages were computed over the number of respondents who provided a valid response for each item.

## 3 Results

### 3.1 Respondents' characteristics

The survey was made publicly available on October 18, 2023 and it was closed on December 31, 2023.

Within this period, a total of 424 adult patients responded to the survey. The majority of respondents (88.7%, *n* = 376) were informed about the survey directly by their clinical center, either through personal contact or email communication. The remaining respondents learned about the survey either through patient associations or scientific societies (5.9%), or voluntarily joined after seeing announcements on social media platforms (5.4%).

Respondents came from diverse Italian regions. See section “3.8 Geographical stratification of survey outcomes” for a complete description.

The gender distribution of respondents was balanced, with 216 males (50.9%) and 206 females (48.6%); 2 respondents preferred not to declare their gender. Respondents were stratified across multiple age groups ranging from 16 to over 85 years, with the majority (*n* = 376; 88.7%) aged between 36 and 84 years. Specifically, 0.4% respondents were 16–17 years old, 8% were between 18 and 35 years old, 26.6% were between 36 and 54 years old, 21.7% were between 55 and 64, 22.1% were between 65 and 74, 18% were between 75 and 84, and 3% were older than 85 years old.

Regarding educational background, 8.7% had completed up to primary school, 24.1% had middle school education, 43.6% had completed up to high school, 23.1% held a bachelor's degree. A small minority (0.5%) held a PhD.

When asked about their comfort with technology, the majority (65.6%) reported being comfortable, while 34.4% reported discomfort or limited confidence in using technological devices.

**Figure 1:**
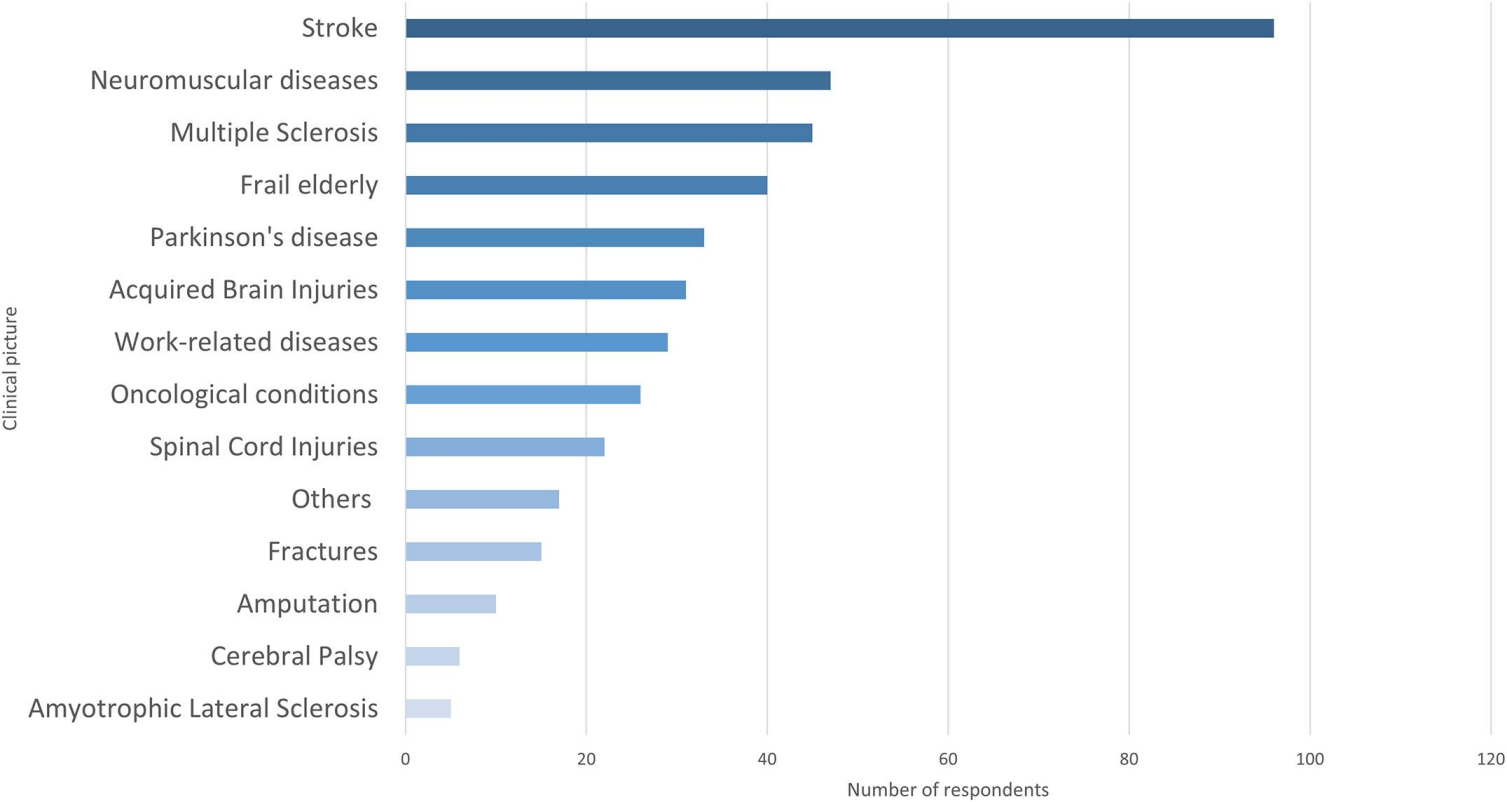
Bar graph of patients’ clinical pictures. The category “Others” includes possible pathologies not attributable to the other mentioned ones but sharing common characteristics with them (e.g., frail elderly, subjects affected by oncological or work-related pathologies).

The clinical conditions targeted by the Initiative were comprehensively represented among respondents (see Figure 1). In this figure, the category “Others”, as mentioned above, includes possible other pathologies not attributable to the mentioned ones but sharing common characteristics with them (e.g., frail elderly, subjects affected by oncological or work-related pathologies).

The most frequently reported condition was stroke (*n* = 96, 22.6%), followed by neuromuscular diseases (*n* = 45; 10.6%) and multiple sclerosis (*n* = 45; 10.6%). Other well-represented categories included frail elderly, Parkinson's disease, and acquired brain injuries. Very few respondents listed, through optional comment boxes, rare or rheumatological conditions or conditions difficult to classify.

**Figure 2:**
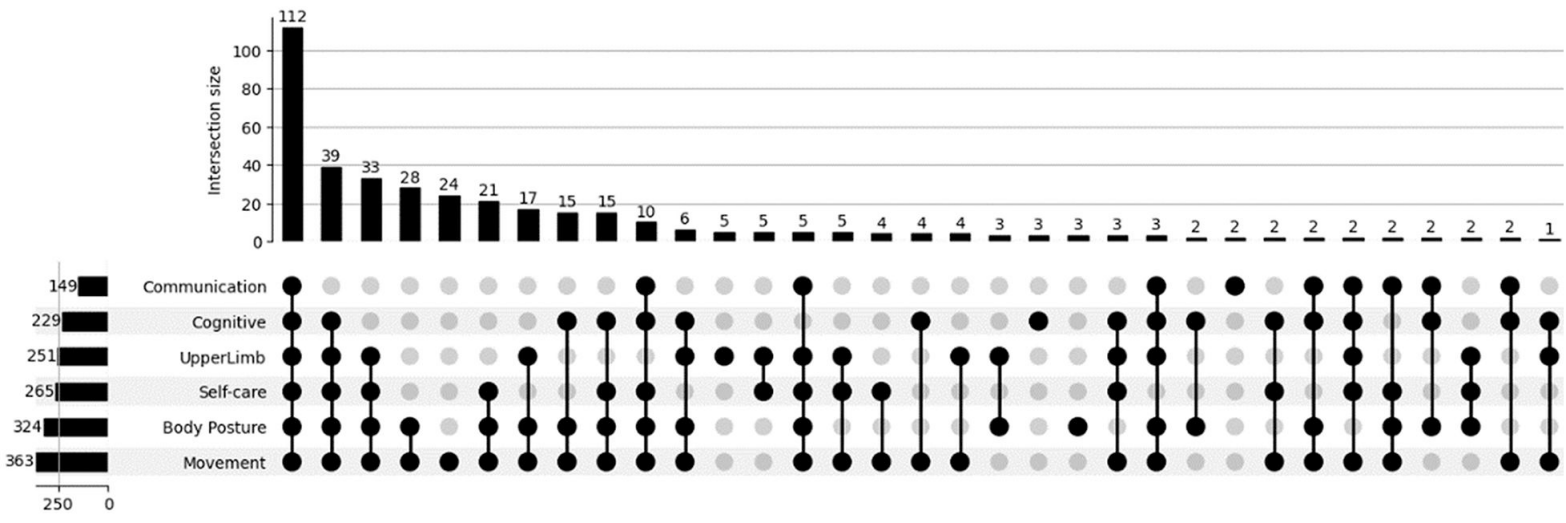
Upset plot showing the number of patients affected in multiple domains. combinations of domains with fewer frequency, considered in the analysis of the survey, have been neglected here for display purposes.

Regarding the impairment of the functional domains explored (namely movement, upper limb function, posture and balance, self-care, cognitive functions, and communication), 26.4% of responders (112 out of 424) presented simultaneous involvement of all functional domains under study, while 9.2% (39 out of 424) presented simultaneous involvement of all domains except communication. Figure 2 displays the distribution of respondents according to the number and type of co-occurring functional impairments, highlighting the most frequent combinations of affected domains.

### 3.2 Rehabilitation modalities

For each functional domain investigated, respondents indicated whether they had undergone rehabilitation targeting deficits in that domain over the past year, specified the rehabilitation modality (traditional, robotic, or both), and reported their level of satisfaction.

Among all respondents, 38.6% (*n* = 164) reported having received treatment using assistive or rehabilitative technologies targeting at least one domain. Of them, 31% reported having received robotic rehabilitation daily, 44% two or more than two times a week, 25% less than two times a week. Instead, 86.1% (*n* = 365) of respondents reported having received traditional treatment over the last year for at least one of those domains. Of them, 34% reported having received traditional rehabilitation daily, 45% two or more than two times a week, 21% less than two times a week. Fifty-one respondents (12%) did not undergo rehabilitation in the last year, neither traditional nor technological.

**Figure 3:**
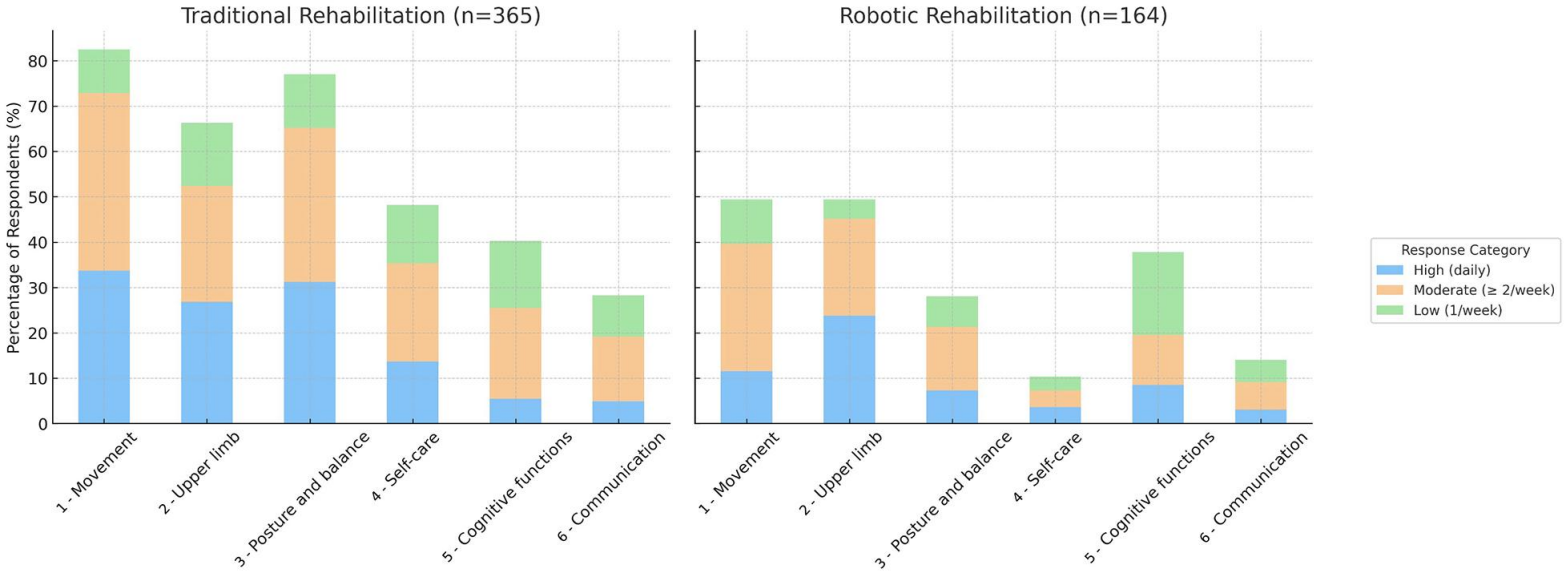
Frequency of rehabilitation treatment across the six functional domains under study, separately for traditional (left) and robotic (right) rehabilitation. The data are expressed as percentages of the total number of respondents for each treatment type (*n* = 365 for traditional, *n* = 164 for robotic). Blue: daily treatment; orange: more than two times per week; green: at most one time a week.

Figure 3 illustrates the distribution of treatment frequency (daily, two/more than two times a week, less than two times a week) across the six functional domains, comparing traditional and robotic rehabilitation approaches. The data are reported as percentages of the total number of respondents for each treatment type (*n* = 365 for traditional, *n* = 164 for robotic). As shown, the distribution of responses was sparser for robotic rehabilitation than the traditional one, which may reflect a more specialized and selective use of robotic technologies in clinical practice: patients tend to access to robotic treatments for a specific domain, while traditional rehabilitation is more broadly applied across multiple domains. For traditional rehabilitation, the domains most frequently targeted by daily treatment were movement and posture and balance, followed by upper limb function. For robotic rehabilitation, upper limb function was the most frequently treated domain, with 48.1% of respondents indicating daily use. In proportion to the other domains, robotic rehabilitation for posture and balance was less frequently accessed by respondents during the week than in traditional rehabilitation, while movement showed moderate frequency of robotic treatment. Self-care and communication were the least frequently treated domains through robotics. Cognitive functions were mostly treated once a week.

Respondents also reported on their rehabilitation settings: 63.2% (*n* = 268) were inpatients, 160 respondents undergoing traditional rehabilitation only, 5 respondents undergoing robotic rehabilitation only, and 103 respondents undergoing both; 40.1% (*n* = 170) received rehabilitation as outpatients or in day-hospital, with 166 respondents undergoing traditional and 60 undergoing robotic rehabilitation; 34.4% (*n* = 146) underwent rehabilitation at home, with 138 respondents doing traditional, and 46 doing robotic rehabilitation. Note that 115 responders underwent both inpatient and outpatient treatment, 110 both inpatient and home, 86 both outpatient and home.

**Figure 4:**
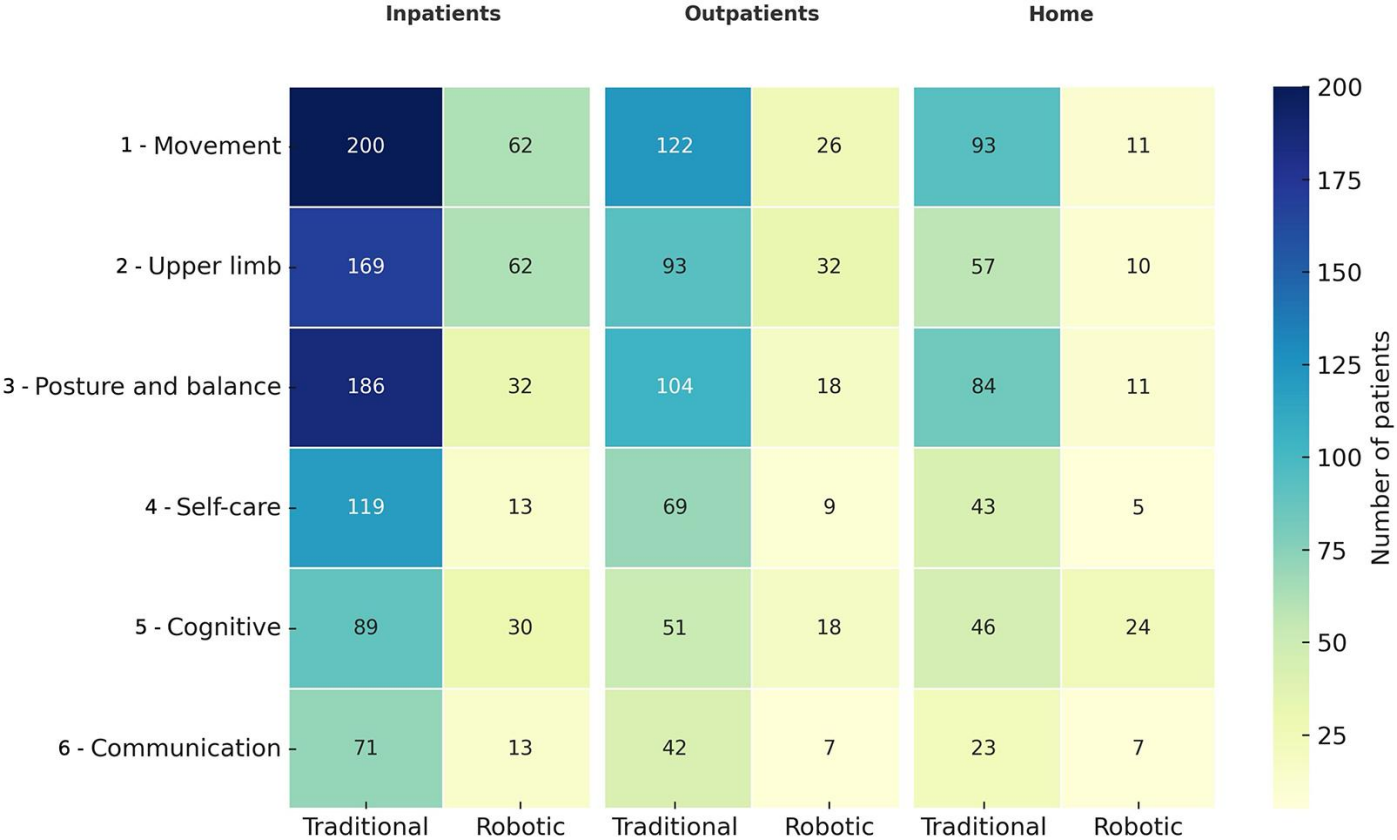
Heatmap showing the number of adult patients who reported having performed traditional or robotic rehabilitation across different clinical settings (inpatient, day hospital/outpatient, home) for each functional domain. The color intensity reflects the number of respondents who declared engaging in the corresponding type of treatment. Note that for each domain, respondents may report both traditional and robotic rehabilitation.

Figure 4 shows the number of respondents who underwent or are undergoing rehabilitative treatment for each domain in relation to the clinical setting. Regardless of the type of rehabilitation, inpatient setting accounted for the largest volume of rehabilitative activity across all domains; the smallest percentage of respondents was instead treated in home settings. In the inpatient setting, movement (*n* = 200, 76.0% of the respondents undergoing traditional rehabilitation), posture and balance (*n* = 186, 70.7%), and upper limb function (*n* = 169, 64.3%) were the most commonly treated domains through traditional approaches, whereas robotic rehabilitation was most frequently used for movement (*n* = 62, 57.4% of inpatients undergoing robotic rehabilitation) and upper limb (*n* = 62, 57.4%). In outpatient and day hospital settings, the trend was similar; however, while traditional therapy most commonly addressed movement (*n* = 122, 73.5% of outpatients receiving traditional rehabilitation), robotic rehabilitation was predominantly applied to the upper limb (*n* = 32, 53.3%).

Communication and cognitive functions were the least involved domains in inpatient and outpatient settings with traditional rehabilitation, while the domains of communication and self-care were performed by fewer respondents through robotics. Home-based rehabilitation showed notably lower frequencies overall, but still highlighted movement (traditional: *n* = 93, 67.4%; robotic: *n* = 11, 23.9%) and posture and balance (traditional: *n* = 84, 60.9%; robotic: *n* = 11, 23.9%) for both modalities, and cognitive for robotics (*n* = 24, 52.2%) as the most commonly reported domains. Self-care and communication were the least treated ones.

**Figure 5:**
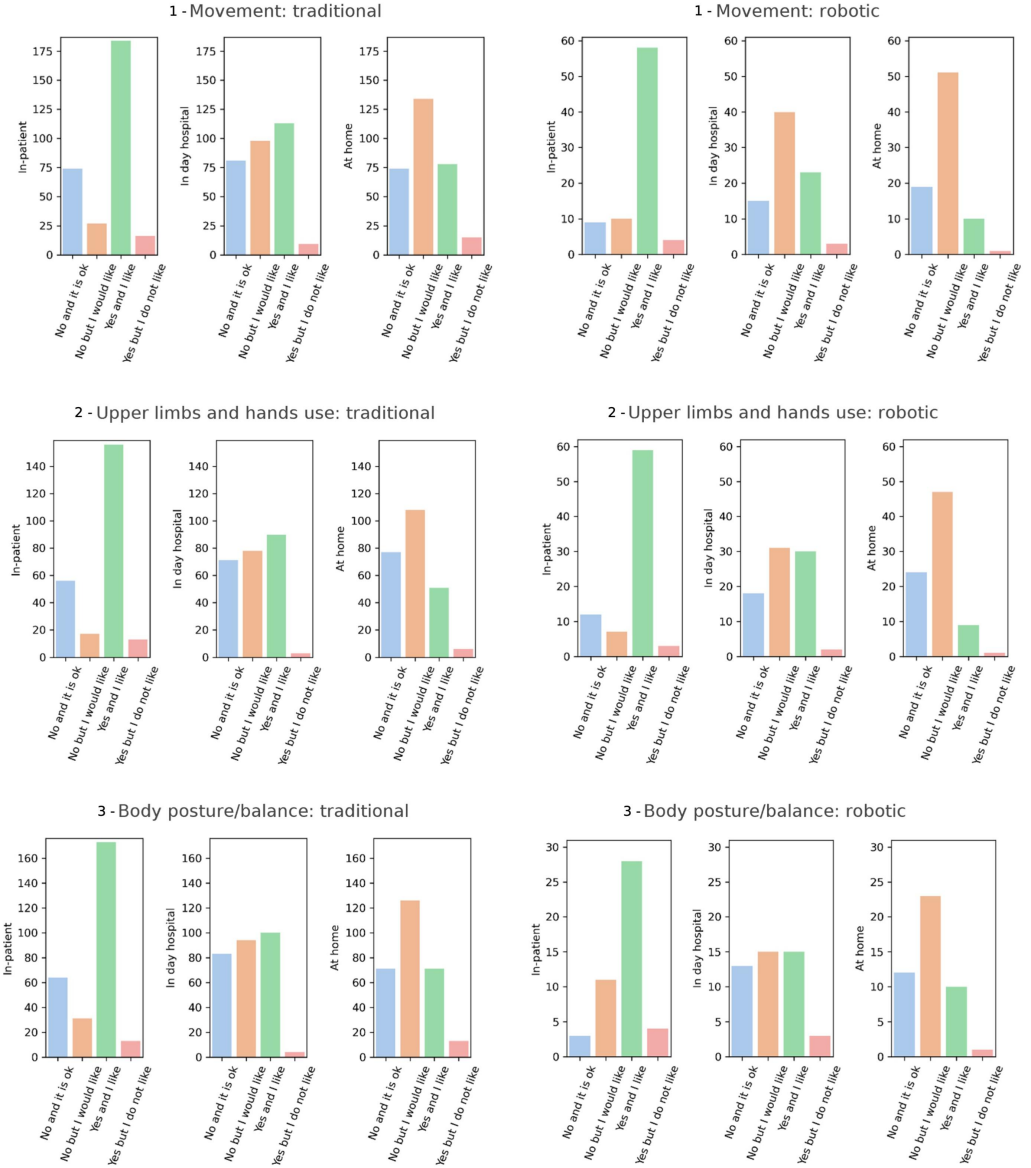
Bar graphs showing, for three relevant settings (inpatients, day hospital, and at home), the number of different answers to the question “Are you currently working, or have you worked in the past year on this specific domain, with a traditional/robotic rehabilitative treatment?”, for all the six functional domains considered in the study.

The bar graphs in Figures 5 depict, for each domain and in relation to the three different settings, how many respondents: (i) did not undergo rehabilitation and were fine with it, (ii) did not undergo rehabilitation but would have liked to, (iii) underwent rehabilitation and were satisfied with it, and (iv) underwent rehabilitation but did not like it. The responses are divided based on the type of rehabilitation treatment (traditional or robotic).

For the *movement* domain (Figure 5, top panels), responses show a clear trend across settings. In the traditional rehabilitation approach, most respondents reported having received treatment and being satisfied with it (“Yes and I like”), particularly in the inpatient setting, where this category reached its peak. However, this percentage progressively decreased in day hospital and home settings, while the proportion of respondents expressing an unmet need (“No but I would like”) increased accordingly, especially at home. The percentage of respondents who underwent rehabilitation but did not like it (“Yes but I do not like”) remained consistently low across all settings. When considering the robotic approach, the absolute numbers were considerably lower across all settings compared to the traditional modality, as mentioned above. Nonetheless, a similar trend emerged: satisfaction (“Yes and I like”) was highest in inpatient settings and diminished in outpatient and home-based contexts, paralleled by a growing proportion of respondents expressing a desire to access robotic rehabilitation (“No but I would like”). Overall, robotic treatment was less frequently reported, but the satisfaction among users appeared relatively high, with minimal dissatisfaction (as we report in the section below). Across all the other domains, this trend was consistent: satisfaction with traditional rehabilitation was highest in the inpatient setting and dropped in home-based scenarios, while the percentage of respondents expressing unmet needs rose; robotic treatment remained marginally used across all settings, with the majority of respondents either not having access but expressing interest, or receiving it and being satisfied with that.

Coherently, when asked whether they were interested in learning more about robotic or technological rehabilitation solutions in relation to the six functional domains, a consistent portion of respondents expressed some level of interest. Specifically, the number of affirmative responses, indicating interest despite not currently using technological tools, ranged from 37.0% of respondents (*n* = 157) for communication to 67.9% (*n* = 288) in the area of mobility. Despite the domain-specific variability in perceived relevance of such technologies, a positive general attitude toward robotic rehabilitation was thus observed.

### 3.3 Satisfaction with rehabilitation treatments

Respondents rated their satisfaction with the traditional and/or robotic rehabilitation they received on a scale from 1 to 5. To evaluate whether overall satisfaction with rehabilitation differed between traditional and robotic approaches, we computed the mean satisfaction score for each patient by averaging their ratings across the six functional domains. Mean satisfaction across responders for traditional rehabilitation was 4.02 (SD = 0.85), while for robotic rehabilitation was 4.08 (SD = 0.88). A non-parametric Mann–Whitney *U* test revealed no statistically significant difference between the means of the two groups (*U* = 28156.5, *P* = 0.265).

The proportion of respondents that were highly satisfied with their treatment (i.e., score ≥4) was however higher for robotic rehabilitation (75.0%) in comparison with traditional rehabilitation (63.8%). Chi-squared tests, conducted to compare the proportion of respondents reporting high satisfaction between traditional and robotic rehabilitation groups, revealed a statistically significant difference in satisfaction for movement rehabilitation [*χ*^2^(1) = 5.433, *P* = 0.020, Cramer's *V* = 0.119]. However, after adjusting for multiple comparisons by using the Benjamini–Hochberg (BH) procedure for controlling the false discovery rate, the BH-FDR adjusted *p* was 0.120. Differences in other domains were not statistically significant, although robotics show higher percentages in all domains. Table 1 reports, for each domain, the percentage and number of respondents with high satisfaction degree for (1) traditional and (2) robotic rehabilitation.

**Table 1:** Satisfaction degree of traditional and robotic rehabilitation across domains.

Domain	Satisfaction degree (≥4) for traditional rehabilitation	Satisfaction degree (≥4) for robotic rehabilitation	*p*-value (BH-FDR adjusted p)
Movement	**74%** **(223/301)**	**86%** **(70/81)**	**0.020** (0.120)
Upper limb	75% (181/242)	84% (68/81)	0.090 (0.270)
Posture and balance	77% (218/281)	78% (36/46)	0.918 (0.918)
Self-care	68% (120/176)	82% (14/17)	0.226 (0.452)
Cognitive	70% (103/147)	77% (48/62)	0.278 (0.417)
Communication	76% (78/103)	78% (18/23)	0.797 (0.956)

*P*-values related to the Chi-squared tests, for each domain, are reported in the last column. Significantly different percentages are reported in bold. Benjamini–Hochberg (BH-FDR) correction for multiple comparisons was applied and reported in brackets.

Stratified analyses of satisfaction levels with respect to gender, age group, geography distribution and education level are reported in Supplementary Materials.

### 3.4 Analysis of functional domains

Beyond the modality of rehabilitation and the satisfaction with it, respondents also evaluated autonomy levels and the impact of their impairment on activities of daily living (ADL) across the six functional domains. Specifically, for each domain, respondents answered two structured questions: one assessing their perceived level of autonomy (“What is your level of autonomy in this domain?”), and one evaluating how much this level of autonomy impacts their ADLs and overall quality of life (“What impact does your level of autonomy in this domain have on your life?”). Here, we report the results related to the single domains separately.

#### 3.4.1 Movement

To explore the relationship between perceived autonomy in movement and its impact on daily life, respondents were asked two questions: (1) “What is your level of autonomy for moving?”, with five answer options ranging from complete independence to complete dependence; and (2) “What impact does the way you move have on your life?”, with five options from “I do not think it is a problem at all” to “I think it is a big problem”.

Among respondents, mobility represented a significant area of impairment. Specifically, 15.6% (*n* = 66) of respondents indicated complete dependency on external assistance for mobility; 14.1% (*n* = 60) answered they move with a device (e.g., electric chair), managing it independently; 27.4% (*n* = 116) answered they can move alone for short distances but prefer to use some aid (e.g., quadripod); 28.5% (*n* = 121) answered they can move on his/her own but handrail is useful to prevent stumbling; 14.4% (*n* = 61) answered they can move on his/her own and even run or jump. Furthermore, 34.2% (*n* = 145) described mobility limitations as a major barrier to their ADLs: 99 respondents (23.3%) indicated that their reduced mobility is a major problem; 46 respondents (10.8%), although not completely dependent on aids, still considered their reduced mobility issues to be severe.

Traditional rehabilitation was the primary intervention for mobility (71.3%, *n* = 302), while robotic rehabilitation was less frequent (19.1%, *n* = 81).

#### 3.4.2 Upper limb function

Upper limb function was notably impaired among respondents, with 18.2% (*n* = 77) considering the upper limbs function as a substantial issue impacting their ADLs. This percentage included 57 respondents (13.4%) who explicitly stated that their autonomy using upper limbs is a major problem, and 20 respondents (4.7%) who, although relying on some adaptations of materials or help from others, still considered their upper limb function challenges to be serious. Furthermore, a notable portion of respondents, 21.5% (*n* = 91), believed that upper limb problems prevent them “from doing some things”. Among these respondents, 38.5% (35 out of 91) answered they can handle many different objects, albeit slowly or with uncertainty and sometimes incomplete precision, while 37.4% (34 out of 91) answered they can only manipulate certain objects in selected situations or that are adapted to the situation (i.e., facilitated).

Traditional rehabilitation for upper limb was common (57.1%, *n* = 242, mean satisfactio*n* = 4.07), and robotic rehabilitation was comparatively less widespread (19.1%, *n* = 81).

#### 3.4.3 Body posture and balance

Postural stability and balance represented a considerable concern among respondents, with 28.1% of respondents (*n* = 119) dependent on posture aids, and 28.3% (*n* = 120) reporting substantial impairment for their ADLs. Specifically, 14.6% (*n* = 62) indicated that their balance and posture impairment is a big problem, and 13.7% (*n* = 58), although not completely dependent on aids (e.g., wheelchairs or canes), still considered their balance and posture issues to be severe.

Traditional rehabilitation was extensively adopted in this domain (66.3%, *n* = 281), whereas robotic interventions were less frequently used (10.9%, *n* = 46).

#### 3.4.4 Self-care

A significant portion of the respondents, 34.9% (*n* = 148), believed that their autonomy for personal care does not pose a problem in their lives. Among these respondents, a substantial majority, 88% (*n* = 130), reported being totally independent. Despite the overall positive perception among many respondents, 22.6% of respondents (*n* = 96) perceived their autonomy for personal care as a substantial issue impacting their daily lives, 14.9% (*n* = 63) experienced severe limitations and complete dependency in daily routines, and 7.8% (*n* = 33), although counting on some adaptations, still considered their self-care challenges to be serious.

Among all the respondents, 42.2% (*n* = 179) declared having rehabilitation with a traditional approach, while only 4% (*n* = 17) had rehabilitation using robotics.

#### 3.4.5 Cognitive functions

Respondents were asked to describe their level of autonomy in performing cognitive tasks and to evaluate how much this level of autonomy impacted their ADLs. A significant portion of the respondents, 50.2% (*n* = 213), believed that their ability to perform cognitive tasks does not pose a problem in their ADLs. Among these respondents, a substantial majority, 84% (*n* = 179), reported being totally independent in executing cognitive tasks. This suggests that they can perform daily cognitive activities without requiring assistance. However, a noteworthy subset of respondents indicated significant difficulties, with 4% (*n* = 17) entirely reliant on caregivers, therefore facing significant cognitive challenges requiring external support, and 13.7% (*n* = 58) considering cognitive impairments a substantial obstacle to daily functioning. Specifically, 5.2% (*n* = 22) explicitly stated that their cognitive difficulties are a major problem, and 8.5% (*n* = 36), although relying on help from others or task simplifications, still considered their cognitive challenges to be serious.

Traditional cognitive rehabilitation was performed by 34.7% (*n* = 147) of respondents, while robotic-assisted approaches were less common (14.6%, *n* = 62). Overall satisfaction levels were relatively similar between rehabilitation modalities: mean satisfaction = 3.96 for traditional, 3.93 for robotic rehabilitation.

#### 3.4.6 Communication

Communication impairments appeared to be less prevalent among respondents, with 70.8% (*n* = 300) reporting minor or no significant problems. Among these respondents, a substantial majority, 87% (*n* = 260), reported being totally independent in communicating, understanding messages and interacting with different people. Nevertheless, 5.2% of total respondents (*n* = 22) answered they depend totally on others for communication or rarely communicate, only with family members, and 6.1% (*n* = 26) of respondents answered they face considerable communication barriers in daily interactions. Specifically, 4.5% (*n* = 19) explicitly stated that their communication difficulties are a major problem, and 1.7% (*n* = 7) although relying on help from some aids, still considered their communication challenges to be serious.

Traditional rehabilitation methods were utilized by 24.3% (*n* = 103) of respondents, achieving a mean satisfaction score of 3.98, whereas robotic rehabilitation was significantly less utilized (5.4%, *n* = 23) but received high mean satisfaction ratings (approximately 4 out of 5).

### 3.5 Treatment priority

Respondents were also asked to rank domains in order of treatment priority (Table 2). Mobility was indicated most frequently as the top priority.

**Table 2:** Ranking of domains in order of treatment priority.

Domain	Number of times indicated as top priority
Movement	160
Upper Limb function	66
Body posture and balance	74
Self-care	34
Cognitive functions	41
Communication	22

**Figure 6:**
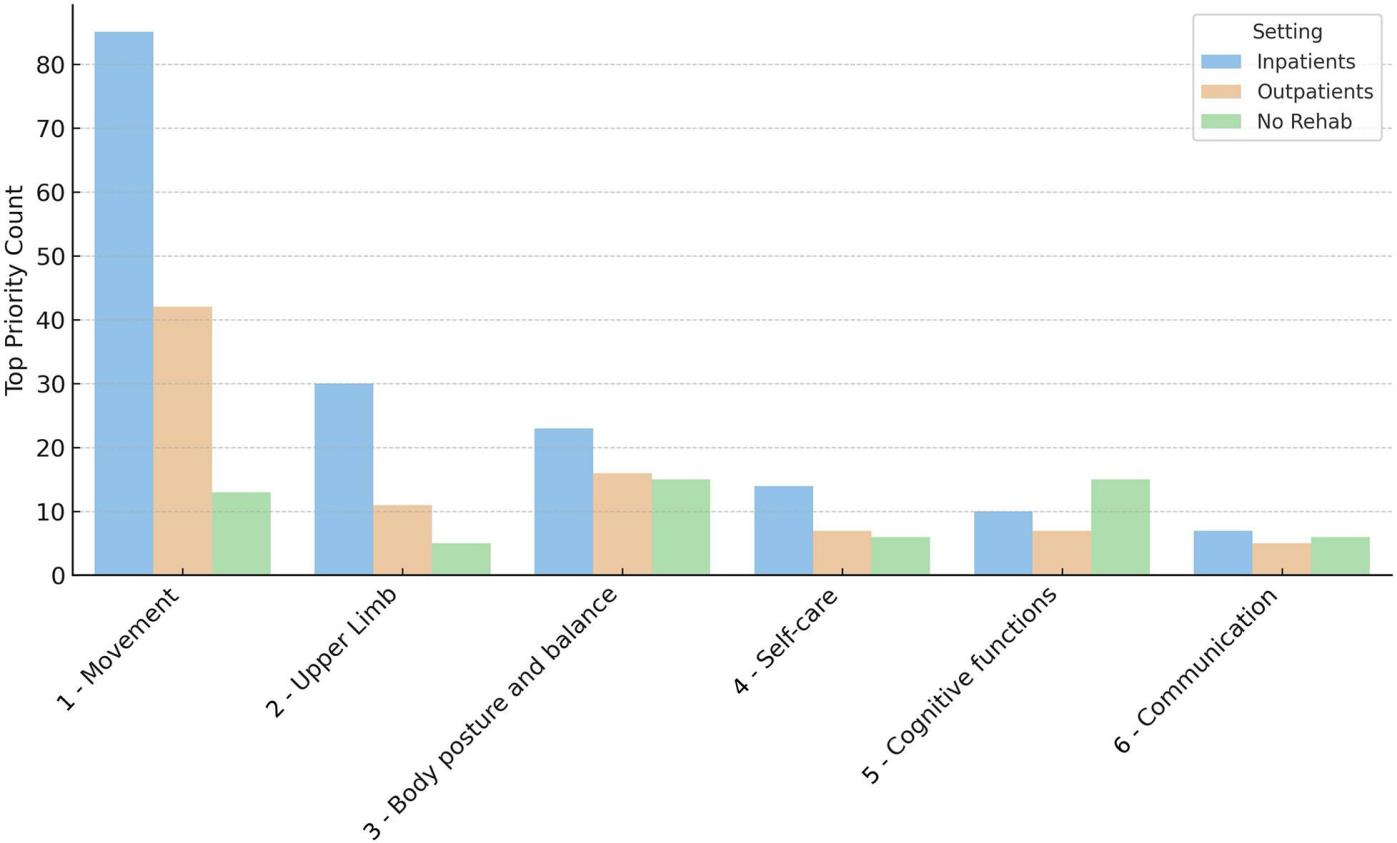
Ranking of functional domains reported as top rehabilitation priorities by patients, stratified by clinical setting. The bar chart shows the number of times each domain (e.g., movement, upper limb function, cognitive functions) was indicated as the most important priority in three patient groups: inpatients (blue), day hospital/outpatients (orange), and patients not currently undergoing rehabilitation (green).

Analysis stratified by clinical setting (see Figure 6) revealed differences in priority needs:

- for inpatients, movement (*n* = 85, 50.3%), upper limb function (*n* = 30, 17.8%), and body posture and balance (*n* = 3, 13.6%) were ranked highest;
- for patients in day hospital or outpatient settings, movement (*n* = 42, 47.7%), body posture and balance (*n* = 16, 18.2%), and upper limb function (*n* = 11, 12.5%) were prioritized;
- patients not receiving rehabilitation (likely to be in a chronic phase of the disease) reported cognitive functions (*n* = 15, 25%) and body posture and balance (*n* = 15, 25%) as primary needs, with movement (*n* = 13, 21.7%) also significantly considered.

These findings emphasize that movement, upper limb function, and balance are consistent priorities in clinical rehabilitation settings, while cognitive functions become significantly important for patients outside structured rehabilitation programs.

### 3.6 Economic burden

Economic aspects related to rehabilitation treatment were also assessed. The majority of respondents (70.3%, *n* = 298) were fully reimbursed by the National Health System (NHS), whereas 27.8% (*n* = 118) were only partially reimbursed. Notably, the rate of partial reimbursement increased (to about 40%) among respondents receiving home-based rehabilitation.

Respondents were further asked about their specific annual expenses related to rehabilitation. Approximately 6.8% of respondents spent more than €2,000 on home environment adaptations over the last year, 17.2% more than €500, and 82.7% less than €500, although this finding must be contextualized with the fact that the majority of respondents were inpatients. Additionally, 21.9% spent more than €500 on rehabilitation technologies and devices. Transportation costs for rehabilitation purposes were notable, with 63.9% spending up to €500 annually, but also 30% spending between €500 and €2,000. A significant percentage of respondents (28.1%) reported annual expenditures between €500 and €2,000 for healthcare interventions specifically related to rehabilitation paid for privately (e.g., specialist medical examinations). Medicines, supplements, or in general pharmaceutical products or special foods not dispensed by the NHS, required between €500 and €2,000 for the 25.7% of respondents. Assistance from non-medical staff (e.g., carers) costed more than €500 for the 16.7% of the respondents (and between €500 and €2,000 for the 10.6%). A total of 16.3% reported an income loss exceeding €2,000, due to lost or reduced work activity, including that of family members or other unpaid caregivers.

**Figure 7:**
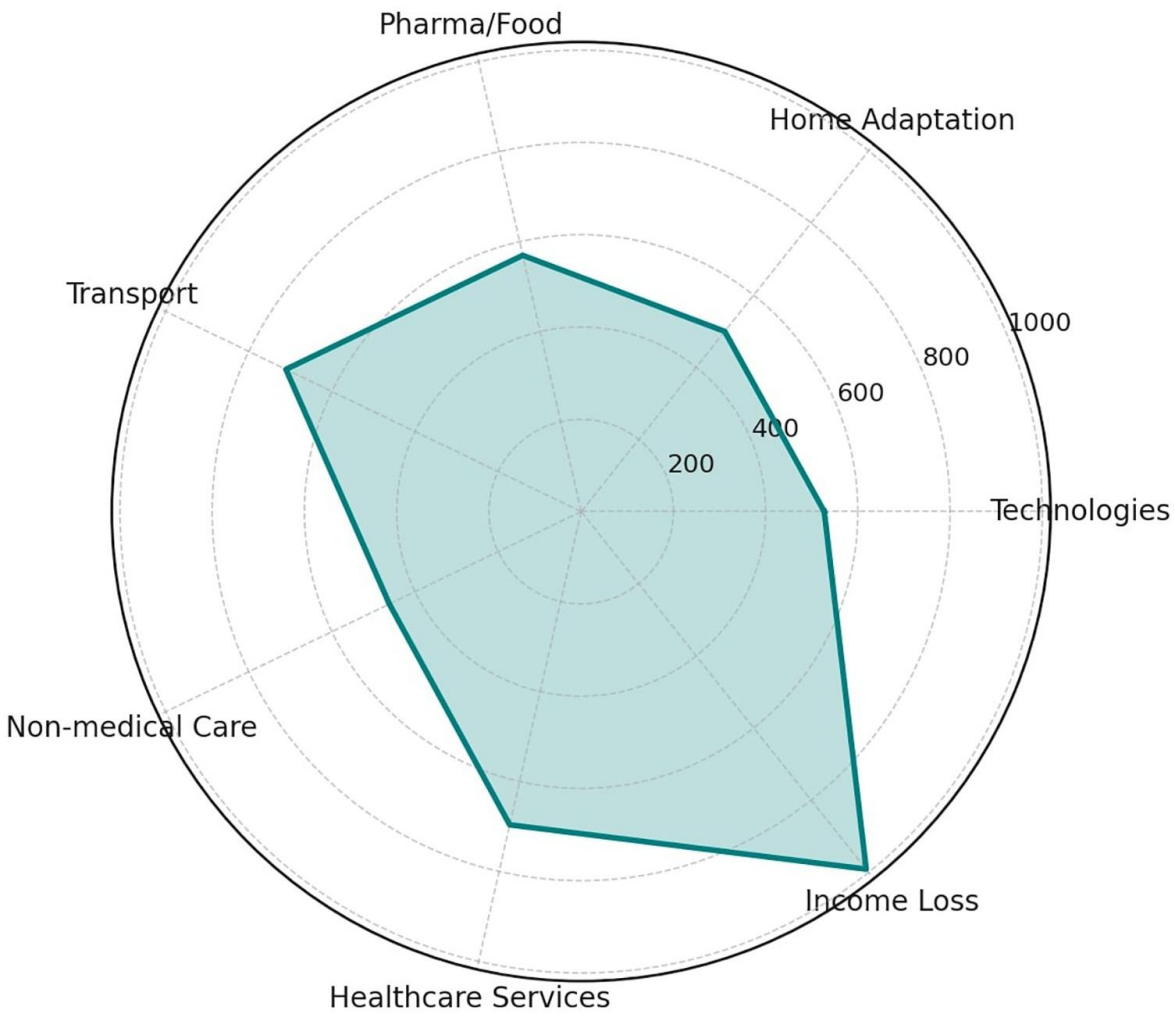
Radar plot showing the estimated average yearly out-of-pocket cost (in €) reported by patients across seven spending categories: technologies, home adaptations, pharmaceutical and dietary products, transport, non-medical assistance, healthcare services, and income loss (see text for details about these items).

Figure 7 shows the estimated economic burden as average yearly out-of-pocket cost, for each of the cost items mentioned above. Average values were computed by assigning mid-range estimates to the ordinal response categories (0 €; <500€; 500€–2,000€; 2,000€–5,000€; >5,000 €).

### 3.7 Qualitative feedback from open-ended responses

Respondents were given the opportunity to freely comment on their rehabilitation experience and express additional needs (*n* = 53 respondents). The qualitative feedback revealed several recurring themes. A substantial number of respondents emphasized the importance of continuity and access to rehabilitation, frequently reporting the inadequacy of public services and the need to pay out-of-pocket for therapies and assistive personnel. Many expressed a strong desire for more frequent and longer physiotherapy sessions, including access to home-based rehabilitation and robotic devices.

Several respondents highlighted the potential of technology to improve autonomy, quality of life, and emotional wellbeing, both through robotic rehabilitation and through daily living aids. Nonetheless, barriers in accessibility, limited information on technological options, and regional disparities were also reported (“I had to drive three hours for good rehabilitation, which should have been available locally.”; “No neuro-physiotherapists in my area. I had to go private.”). Emotional reflections were frequent, with respondents expressing frustration, hope, and the desire to regain independence, particularly in walking and ADLs.

Positive experiences were also documented, especially concerning the professionalism and empathy of rehabilitation staff. However, dissatisfaction emerged when treatment was perceived as interrupted too early or lacking follow-up.

### 3.8 Geographical stratification of survey outcomes

The geographical distribution of survey responses highlighted a predominance of respondents from Northern Italy (52.3%, *n* = 222), followed by the Central regions (33.0%, *n* = 140) and the South (14.6%, *n* = 62). This geographic coverage confirmed the Fit4MedRob Consortium's capability to reach a broad and representative group of disabled adults in Italy. When disaggregating survey outcomes by macro-area, relevant differences emerged.

Access to technological rehabilitation was reported by 32.9% of respondents in the North (*n* = 73/222), 49.3% in the Center (*n* = 69/140), and 35.5% in the South (*n* = 22/62). Access to home-based technological rehabilitation was much less frequent overall, involving only 13.1% of respondents in the North (*n* = 29/222), 9.3% in the Center (*n* = 13/140), and 6.5% in the South (*n* = 4/62). Regarding patient-reported outcomes, satisfaction with technological rehabilitation (score ≥4) was expressed by 23.4% of respondents in the North (*n* = 52/222), 45.7% in the Center (*n* = 64/140), and 30.6% in the South (*n* = 19/62). Interest in learning more about robotic and technological solutions was widespread, although with relevant geographical variability: 78.8% in the North (*n* = 175/222), 85.0% in the Center (*n* = 119/140), and 46.8% in the South (*n* = 29/62). Finally, the estimated annual out-of-pocket expenditure (i.e., the total economic burden) also differed across areas. Higher mean costs were observed in the Center (€5,138, with *n* = 137 respondents reporting expenses >0) and in the North (€4,523, *n* = 208 with expenses >0), compared to the South (€2,726, *n* = 54 with expenses >0).

**Figure 8:**
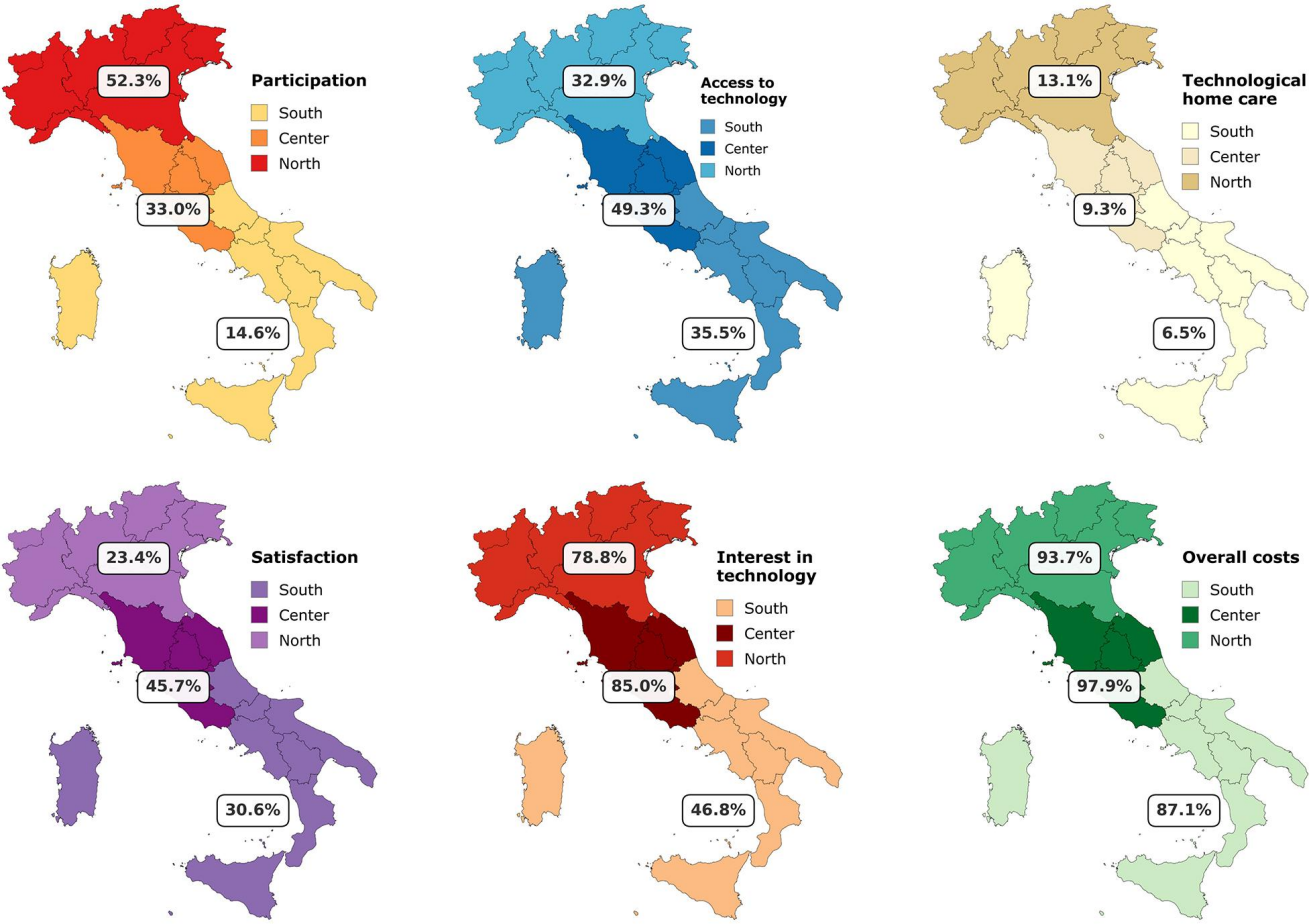
Geographic distribution of survey respondents and key indicators related to technological rehabilitation. Maps illustrate data aggregated by Italian macro-areas (North, Center, South). Participation: proportion of respondents by macro-area. Access to technology: respondents who received technology-based rehabilitation. Technological home care: respondents who received it at home. Satisfaction: respondents reporting high satisfaction with technological rehabilitation (score ≥4) for at least one domain. Interest in technology: respondents expressing interest in learning more about technological rehabilitation solutions in at least one domain. Overall costs: proportion of respondents reporting annual out-of-pocket expenses >0 for rehabilitation. Apart from Participation, all other percentages are relative to the number of respondents within each macro-area.

Collectively, these findings confirm a broad representativeness of the Italian territory, while also underlining macro-regional disparities in technological access, satisfaction, and economic burden (Figure 8).

## 4 Discussion

This study aimed to depict the current rehabilitative scenario in Italy, identify patients' rehabilitative needs and experiences with care, and explore their opinions and attitudes toward robotics and digital technologies in rehabilitation; overall, findings from 424 respondents indicated persistent geographical and economic disparities in access to services, a strong multidomain rehabilitation need with motor function as a primary priority, limited but positively evaluated exposure to robotic interventions, and substantial interest in technology-supported, including home-based, rehabilitation solutions.

Our survey represents the first large-scale attempt in Italy to systematically capture the rehabilitative needs, perceptions, and experiences of adult patients with motor, sensory, and/or cognitive disabilities regarding the adoption of technological and robotic solutions in clinical practice. Building upon the methodology developed in a previous work (18) and guided by the ICF framework, we were able to gather insights from a broad and heterogeneous patient population across multiple pathologies and rehabilitation settings.

The sample of adult patients completing the survey represented a wide range of neurological and musculoskeletal conditions including stroke, multiple sclerosis, Parkinson's disease, neuromuscular diseases, acquired brain injury, spinal cord injuries, amputations, and other neuromuscular or cognitive impairments. The patients were recruited from 12 clinical centers across Italy, ensuring geographical representativeness, i.e., reaching a substantial and representative sample of disabled adults nationwide. Differences between northern and southern regions involvement may be explained by the fact that the clinical centres participating in the survey are predominantly located in northern Italy.

Beyond participation, our findings highlighted relevant geographical disparities not only in the distribution of respondents but also in several key aspects of technological rehabilitation. Given the well-established structural and organizational differences between Northern, Central, and Southern Italy in healthcare resource allocation and service provision, the observed regional disparities in economic burden and access to technological rehabilitation should be interpreted as highly relevant to accurately depict the current rehabilitative scenario in Italy. In Southern Italy, access to technological rehabilitation was relatively good when considered against the number of respondents in that macro-area. However, the availability of technology-based home care was markedly lower compared to the North and Center, suggesting that advanced solutions are less integrated into community and domestic care pathways. The economic burden was generally lower in the South in terms of average costs, yet the proportion of respondents reporting out-of-pocket expenses remained high, underscoring that financial barriers are still present even where expenditure levels are lower. Satisfaction with technological rehabilitation among those who received it was positive across all macro-areas. Nevertheless, interest in learning more about robotics and advanced technologies was significantly lower in the South, indicating greater skepticism or resistance among individuals who have not yet had access to such treatments. Taken together, these findings suggest that disparities in Italy are not only structural and organizational but also perceptual and cultural. Precisely for this reason, the Fit4MedRob Initiative has strategically engaged clinical centers in southern regions, with the aim of progressively reducing the technological gap, overcoming economic and cultural barriers, and fostering equity in the adoption of robotic rehabilitation across the country (9).

Most respondents were comfortable with technology use, and a substantial portion (about 30%) reported receiving inpatient or day-hospital rehabilitation. Results suggest that, at least within the Italian context, adult patients are ready and willing to engage with technological rehabilitation when appropriate solutions are provided. However, a significant number of individuals lacked access to rehabilitation services (about 2%) provided by NHS or received only partial reimbursement for their treatments (about 28%).

Our findings also revealed several key aspects that must be considered to ensure patient-centered integration of rehabilitation technologies.

First, a strong need for multidomain rehabilitation emerged. Currently, robotic interventions appeared focused on specific functional domains, particularly upper limb rehabilitation, indicating growing interest and deployment of robotic systems in this domain. In fact, mobility was identified as the highest treatment priority by the majority of respondents, confirming that motor recovery (lower and upper limb) remains a fundamental target across different neurological conditions. However, significant impairments were also reported in other functional domains, such as self-care and cognitive functions, indicating that rehabilitation technologies should be designed to address a comprehensive range of functional limitations rather than focusing narrowly on motor performance.

Second, despite the increasing interest in robotic rehabilitation, the survey highlighted limited exposure and access to such technologies: only 39% of respondents reported having experienced robotic interventions. This confirms the persistence of technological barriers within clinical settings and underlines the urgent need for broader dissemination and equitable access to robot-assisted rehabilitation, especially considering the high satisfaction levels reported by respondents who used these technologies.

Interestingly, the number of respondents reporting high satisfaction was greater for robotic rehabilitation than traditional rehabilitation. This indicates that, when available, such robotic solutions are perceived as both effective and well accepted by patients. This finding is in line with those reported in (25), even though this study involved a small number of patients (*n* = 12) following spinal cord injury. Sørensen and Poulsen found that both patients and physiotherapists acknowledged the potential of robotic technologies, despite the technical and organizational demands. The qualitative meta-synthesis by Laparidou et al. (13) highlighted recurring themes across motor rehabilitation robotics, including perceived benefits (e.g., engagement, structured practice, and feedback) alongside concerns that can modulate acceptability and sustained use, such as the need for adequate support, usability issues, and integration into routine care pathways. In parallel, evidence focusing on motivation and satisfaction during technology-assisted rehabilitation, including robot-assisted interventions (26), reinforces that acceptability is tightly linked to how technologies shape patients' perceived engagement, autonomy, and meaningfulness of practice, factors that plausibly underpin the high satisfaction rates observed in our cohort. Notably, no significant differences in satisfaction emerged in our study between traditional and robotic rehabilitation in the cognitive and communication domains. On the one hand, this suggests that robotics does not negatively affect patients' satisfaction; on the other hand, it highlights the need for further research and the development of robotic systems specifically designed to address these areas.

Therefore, while high satisfaction and acceptability are necessary conditions for successful translation (27, 28), health-service implementation suggests they are not sufficient. Our and previous surveys' evidence in rehabilitation contexts (4, 5) has shown that organizational and implementation constraints (e.g., workflow integration, training, and resource availability) can limit real-world uptake even when technologies are considered promising by end users, a concept that is well-known in international literature on implementation science (29). Interpreted through this lens, our findings support a two-level narrative: patients often perceive robotic rehabilitation positively, consistent with qualitative evidence, yet broader adoption requires implementation strategies that address service-level constraints (training, workflows, and sustainability) in addition to clinical effectiveness (9).

Another crucial aspect that emerged concerns the need for home-based rehabilitation. As pointed out by professionals in the study of (4), home-based technologies for rehabilitation have great potential to facilitate accessibility, autonomy, and increase choice for therapy. As a matter of fact, a substantial proportion of our respondents, undergoing rehabilitation in clinical settings, reported difficulties in accessing rehabilitation services, along with relevant income loss and out-of-pocket expenses: home adaptations and transport emerged as the most burdensome categories, highlighting the patient's side costs associated with long-term rehabilitation. Moreover, among home-based respondents, a higher rate of partial reimbursement by the NHS was noted. These findings strongly advocate for the development of accessible and sustainable home-based robotic rehabilitation programs, which could alleviate economic burdens and promote continuity of care.

This result is coherent with implementation-oriented evidence showing that resource limitations and financial constraints are recurring obstacles to deploying robotic technologies in healthcare settings (30). We have previously identified costs and resource-related constraints among key barriers to robotic implementation in clinical care (9), suggesting the need for implementation strategies that explicitly address these dimensions. In rehabilitation robotics specifically, health-economic reviews and analyses highlight that acquisition and operational costs, reimbursement, and the broader economic sustainability of robotic programs remain central considerations for adoption (31, 32).

Taken together, our findings support the view that translation of rehabilitation robotics is not driven solely by efficacy but by a combination of patient experience, clinical workflow integration, staff training, and economic sustainability. The convergence between patient-reported economic barriers in our survey and barriers reported in implementation and professional-user studies suggests that future work should incorporate health-services research approaches (e.g., implementation outcomes and economic evaluations) alongside clinical effectiveness to inform scalable service models.

Within this perspective, the multi-domain structure adopted in our survey can be viewed as a patient-centered contribution to IRP development, providing information on priorities and perceived gaps that may inform ICF-based goal selection and personalization of treatment content (including robotic-assisted interventions) across settings. At the same time, policy-oriented reflections on rehabilitation systems emphasize that the IRP should not be considered in isolation from the broader service context. Specifically, the feasibility of translating individualized goals into practice depends on organizational and environmental factors, including access, resources, and system constraints (33).

As part of the Fit4MedRob Initiative, this work aims to inform and guide the design of pragmatic clinical trials focusing on the efficacy and sustainability of robotic rehabilitation, the results of which will suggest approaches to overcome the above-mentioned barriers to the adoption of robotics and allied digital technologies in rehabilitation (34–36). By systematically capturing real-world patient needs across multiple domains, this survey ultimately provides a critical foundation for ensuring that future rehabilitation technologies are both effective and truly user-centered.

### 4.1 Limitations

The survey was subject to potential sampling bias, as participation was voluntary and may have attracted patients more engaged with technology or rehabilitation processes. Second, non-response bias cannot be excluded, particularly regarding specific clinical subgroups that were underrepresented. Furthermore, self-reported information from the portion of respondents recruited through the rehabilitation centers where they received care, potentially may have increased the tendency to provide favorable evaluations, and results may thus be affected by social desirability or acquiescence effects. Mitigations to this issue are that participation was voluntary, responses were anonymous, and recruitment also involved patient associations. Third, as the target population includes neurological conditions with possible cognitive impairment, the absence of formal cognitive screening may have influenced comprehension and response accuracy in a subset of respondents. The questionnaire was designed to be concise and accessible, and a parallel caregiver-focused survey was conducted within the Fit4MedRob initiative to capture perspectives for patients who may not be able to respond autonomously.

Fourth, our sample included multiple neurological and motor conditions with potentially different rehabilitation needs and priorities. Although aggregated analyses allow identification of cross-cutting themes relevant to service organization and technology implementation, diagnosis-specific patterns may differ substantially and cannot be inferred from pooled results. Future work will therefore examine pathology-stratified subgroups and/or condition-specific cohorts to assess whether priorities, satisfaction and attitudes toward robotics and digital technologies vary by diagnosis.

Finally, the cross-sectional nature of the survey precludes drawing conclusions about the evolution of needs and experiences over time, which will be matter of future studies and pragmatic clinical trials.

The survey was conceived as a stakeholder needs-assessment instrument aimed at informing clinical trial planning rather than as a finalized psychometric scale. Consequently, while content validity was addressed through ICF-based mapping and iterative stakeholder review, formal psychometric validation of the proposed methodology (e.g., test–retest reliability, intra-/inter-rater reliability on non-anonymous completions) was not conducted within the present study. Additionally, future research should incorporate more in-depth qualitative methodologies to comprehensively investigate patients' trust in robotic systems, perceived safety, emotional responses, and other experiential dimensions of robotic rehabilitation.

## 5 Conclusions

This study provides a comprehensive overview of the rehabilitation needs and priorities of adult patients with neurological and neuromuscular conditions in Italy. The findings highlight the demand for multidomain, accessible, and home-based rehabilitation interventions, as well as the limited diffusion but high perceived value of robotic-assisted therapies. These insights, grounded in the ICF framework, offer valuable guidance for clinicians, developers, and policymakers aiming to align future technologies with patient-centered care. The results will inform the Fit4MedRob Initiative's ongoing efforts to design pragmatic trials and support the development of effective, sustainable, and inclusive rehabilitation solutions.

## Data Availability

The raw data supporting the conclusions of this article will be made available by the authors, without undue reservation.

## Footnotes

^1^ https://www.fit4medrob.it

## Mission 1 Fit4MedRob Consortium

- Francesca Falchini, Laura Cortellini, Stefania Lattanzi, Arianna Pavan (IRCCS Fondazione Don Carlo Gnocchi ONLUS, 50143 Florence, Italy)
- Enea Parimbelli (Department of Electrical, Computer and Biomedical Engineering, University of Pavia, 27100 Pavia, Italy)
- Emanuele Gruppioni, Francesca Bugané (Centro Protesi INAIL, Istituto Nazionale Assicurazione Contro Gli Infortuni Sul Lavoro, Bologna, Italy)
- Anna Estraneo, Alfonso Magliacano, Rita Mosca (IRCCS Fondazione Don Carlo Gnocchi ONLUS, 50143 Florence, Italy)
- Francesca Cecchi, Tommaso Ciapetti, Michele Piazzini, Chiara Castagnoli, Stefano Giuseppe D'Oronzio (IRCCS Fondazione Don Carlo Gnocchi, Firenze)
- Francesca Baglio, Federica Rossetto (IRCCS Fondazione Don Carlo Gnocchi ONLUS, 20148 Milan, Italy)
- Michele Lategana (IRCCS Fondazione Don Carlo Gnocchi ONLUS, 50143 Florence, Italy)
- Stefano La Rocca (IRCCS Fondazione Don Carlo Gnocchi ONLUS, 50143 Florence, Italy)
- Roberto De Icco, Luca Martinis, Ilaria Campese, Sabrina Signorini, Roberta Nicotra (Movement Analysis Research Section, IRCCS Mondino Foundation, 27100 Pavia, Italy)
- Giuseppe Umile Longo, Arianna Carnevale, Carla Antonacci, Federica Bressi, Ludovica Di Curzio, Lucrezia Giorgi (Fondazione Policlinico Universitario Campus Bio-Medico, 00128, Rome, Italy)
- Pietro Fiore, Brigida Minafra, Stefania De Trane (Istituti Clinici Scientifici Maugeri IRCCS, Neurorehabilitation Unit of Bari Institute, 70124 Bari, Italy)
- Antonio Nardone, Chiara Pavese (Istituti Clinici Scientifici Maugeri IRCCS, Centro Studi Attività Motorie (CSAM) and Neurorehabilitation and Spinal Units of Pavia Institute, 27100 Pavia, Italy)
- Cira Fundarò (Neuropathophysiology Unit of Montescano Institute, Istituti Clinici Scientifci Maugeri IRCCS, Montescano, Pavia, Italy)
- Christian Lunetta, Rachele Piras, Matteo Gallotta (Istituti Clinici Scientifici Maugeri IRCCS, Neurorehabilitation Department, Institute of Milan, Milan, Italy)
- Chiara Maria Tacchino, Alessia Aiello (Physical Medicine and Rehabilitation Unit, IRCCS Istituto Giannina Gaslini, Genoa, Italy)
- Emilia Biffi, Fabio Storm, Eleonora Diella (Scientific Institute, IRCCS Eugenio Medea, Bosisio Parini, Lecco, Italy
- Mehrnaz Hamedani, Angelo Schenone, Matteo Pittaluga, Carlo Trompetto (Division of Neurorehabilitation, IRCCS Ospedale Policlinico San Martino, Genova, Italy)
- Giovanni Comandè, Francesca Gennari (Sant'Anna School of Advanced Studies, 56127 Pisa, Italy)
- Francesca Benuzzi, Jessica Mandrioli, Giovanna Zamboni (University of Modena and Reggio Emilia, Modena, Italy)
- Carlo Ruosi, Rossana Gnasso, Angela Palomba (University Federico II of Naples, Naples, Italy)
- Carmelo Chisari, Stefania Dalise, Valentina Azzollini (University of Pisa, Pisa, Italy)
- Franco Molteni, Alessandro Specchia, Giulio Gasperini, Eleonora Guanziroli (Centro di Riabilitazione “Villa Beretta”, Ospedale Valduce, 23845 Costa Masnaga, Italy)
- Ennio Ferlazzo, Flavio Corpina (C.O.T. Cure Ortopediche Traumatologiche s.p.a, Messina, Italy)
