# Supplementary material for: Identifying needs in adult rehabilitation to support the clinical implementation of robotics and allied technologies: an Italian national survey
Source: Front Digit Health. 2026 Apr 1;8:1718274. doi: 10.3389/fdgth.2026.1718274 (PMC13081780; doi:10.3389/fdgth.2026.1718274)
Supplement: Supplementary file 1 [file Datasheet1.docx]

**Identifying Needs in Adult Rehabilitation to Support the Clinical Implementation of Robotics and Allied Technologies: An Italian National Survey**

Here we present the survey developed as part of the Italian Initiative “Fit for Medical Robotics” (Fit4MedRob)^[[1]](#footnote-1)^ aiming at collecting the perspectives and needs of patients with motor and/or cognitive impairments about the neuromotor and cognitive rehabilitation and the relative adoption of robotics and allied technologies in the field. The survey is translated from the Italian language.

# Fit for Medical Robotics Adult Patients Survey – English version

## Introduction

** Mandatory*

*Welcome to this survey and thank you for your participation!

We are collecting opinions from patients with various clinical conditions to identify, through an anonymous survey, the primary rehabilitation needs in relation to disability. The survey is conducted as part of the FIT4MedRob* project, which aims to experiment with robotics in order to lay the groundwork for the development of increasingly advanced technological systems in the field of rehabilitation.

This questionnaire is anonymous and voluntary.
In the following questions, you will be asked to respond based on the rehabilitation treatment you are currently undergoing or have received in the past year. These questions are divided into six different sections, corresponding to functional domains* *(mobility, posture maintenance, cognitive abilities, communication, self-care and use of upper limbs), each of which is briefly described at the beginning of the section.*

*As you will see, you will be asked to respond regarding two types of rehabilitation interventions: traditional (e.g., with a physiotherapist) and those carried out using technology. This is not intended to assess the effectiveness of the treatments, but rather to understand the national situation regarding the type and quantity of rehabilitation treatment provided.

At the end of the questionnaire, you will have the opportunity to add comments, suggestions, or any feedback you wish to share with us.

Estimated completion time: between 10 and 30 minutes. Please respond by December 31st, 2023, closing date of this survey.

(*More information on the project is available at: https://www.fit4medrob.it)*

*1 Would you like to take part in this survey? **

- *Yes*
- *No*

*2 Please tell us, how did you find out or how were you contacted for this survey? **

- *Directly or by e-mail from the clinical centre I am referred to*
- *Patient associations or scientific societies*
- *I joined voluntarily because I saw the post on social media channels*

*I declare that I have read the information notice *.*

*The information can be found at the following link https://unipiit-my.sharepoint.com/:b:/g/personal/a029218_unipi_it/EZWNlg1oaUdAsJywBY-Cb7YBM3M1JmlxZNrLKknjYlUj9Q?e=85j1Zo*

- *I confirm*

## General Questions

*This questionnaire is anonymous; however, we will ask you some personal questions (e.g., age group or pathology) to be able to analyse all answers by groups of patients with similar characteristics.*

*3 Could you tell us your gender? **

- *Male*
- *Female*
- *I prefer not to declare it*

*4 Could you tell us which age group you belong to? **

- *16-17 years*
- *18-35 years*
- *36-54 years*
- *55-64 years*
- *65-74 years*
- *75-84 years*
- *>85 years*

*5 What is your level of education **

- *Elementary school*
- *Middle school*
- *Secondary school*
- *Master’s Degree*
- *PhD*

*6 In general, could you indicate how comfortable are you with the use of technology? **

- *Not at all*
- *A little*
- *Fairly*
- *Very much*
- *Completely*

*7 What is your clinical picture? **

- *Stroke outcomes* *(How long has it been since the event? a) more than 6 months; b) less than 6 months)*
- *Multiple sclerosis*
- *Acquired brain injury (How long has it been since the event? a) more than 6 months; b) less than 6 months)*
- *Myelolesion (How long has it been since the event? a) more than 6 months; b) less than 6 months)*
- *Outcomes of oncological surgery*
- *Parkinson's disease*
- *ALS*
- *Amputation*
- *Neuropathy*
- *Dystrophy*
- *Cerebral Palsy*
- *Other (Please provide us with some details that will help us understand better)*

*8 Could you tell us your treatment setting, i.e., where he/she receives treatment? **

- *Inpatient regimen*
- *Day hospital or outpatient*
- *I’m currently under the care of the National Health Service (NHS), but I am also undergoing some rehabilitation treatments privately*
- *I’m not currently on the NHS, but I am undergoing all rehabilitation treatments privately*
- *I’m not undergoing rehabilitation treatment*

*9 Which region does the patient refer to in his/her treatment pathway? **

- *Abruzzo*
- *Basilicata*
- *Calabria*
- *Campania*
- *Emilia Romagna*
- *Friuli Venezia Giulia*
- *Lazio*
- *Liguria*
- *Lombardia*
- *Marche*
- *Molise*
- *Piemonte*
- *Puglia*
- *Sardegna*
- *Sicilia*
- *Toscana*
- *Trentino Alto Adige*
- *Umbria*
- *Valle d’Aosta*
- *Veneto*

## Movement domain

*This section refers to movement in the sense of walking, walking forwards, backwards or sideways, walking on different surfaces, indoors or outdoors; getting around or over obstacles, going up/down-stairs, climbing, running, and jumping. This includes movements made using specific devices designed to facilitate movement such as electronic or manual wheelchairs, walkers or quadripods/sticks, etc.*

*10 What is your level of autonomy in movement? **

- *I only move with the help of someone (e.g., who pushes my wheelchair)*
- *I move with a device that I manage autonomously (e.g., electric/electronic wheelchairs)*
- *I can move alone for short distances but I prefer to use some aid (e.g., walker, quadripods, sometimes wheelchair)*
- *I move on my own, even on stairs, but the handrail can be useful, I sometimes stumble*
- *I can move alone, even on stairs, I can run and jump without hesitation*

*11 What impact does the way you move through your life have? **

- *I think it's a big problem*
- *It prevents me from doing certain things and I am sorry about it*
- *Although I can rely on some aids (e.g., wheelchair or quadripods), it is a big problem*
- *It's a small problem because I have functional solutions (e.g., wheelchair or quadripods)*
- *I think it is not a problem at all*

*12 Are you working or have you worked in the last year on this function with a traditional rehabilitation treatment (i.e., without using technological solutions)? **

- *Not at all and that's OK*
- *Not at all but I would like to work on it*
- *Partially (e.g., no more than once a week) ^#^*
- *Fairly (2 or more times a week) ^#^*
- *Very much (daily) ^#^*

*^#^13 How satisfied are/were you with this type of treatment? **

*not at all
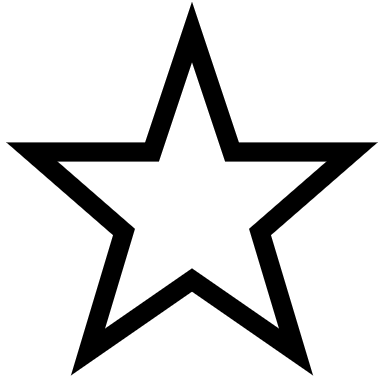

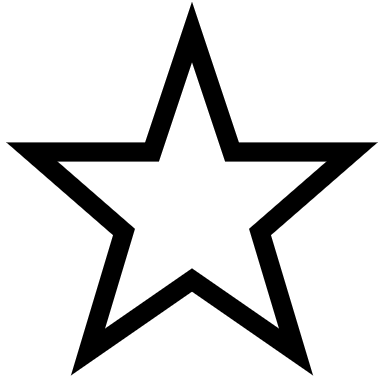

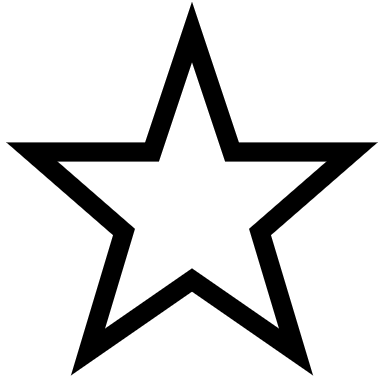

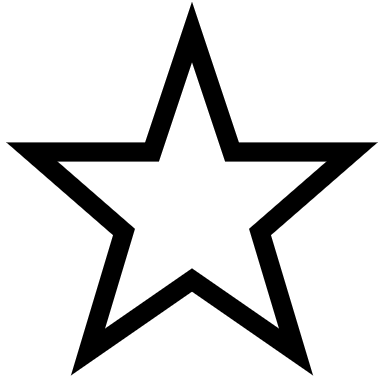

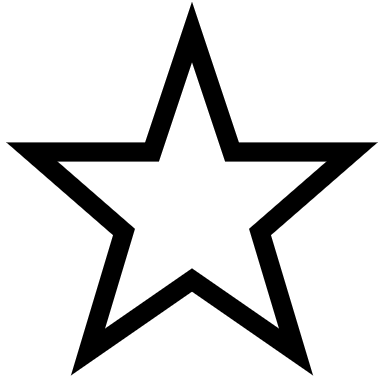
 completely*

*^#^14 Are you performing or have you performed a traditional rehabilitation treatment for this function within a clinical (inpatient) setting?*

- *No, and I'm fine with that*
- *No, but I would like to*
- *Yes, but I don't like it*
- *Yes, and I like it*

*^#^15 Are you performing or have you performed a traditional rehabilitation treatment for this function in an outpatient clinical setting (in day hospital)?**

- *No, and I'm fine with that*
- *No, but I would like to*
- *Yes, but I don't like it*
- *Yes, and I like it*

*^#^16* *Are you performing or have you performed a traditional rehabilitation treatment for this function at home? **

- *No, and I'm fine with that*
- *No, but I would like to*
- *Yes, but I don't like it*
- *Yes, and I like it*

*17 Are you working or have you worked in the last year on this function with a rehabilitation/assistive treatment involving technology? (an example would be an exoskeleton for walking) **

- *Yes^#^*
- *No*

*^#^18 How much are you working or have you worked in the last year on this function with a rehabilitation/assistive treatment involving the use of technology? **

- *Partially (e.g., no more than once a week)*
- *Fairly (2 or more times a week)*
- *A lot (every day)*

*^#^19 How satisfied are/were you with this type of treatment? **

*not at all
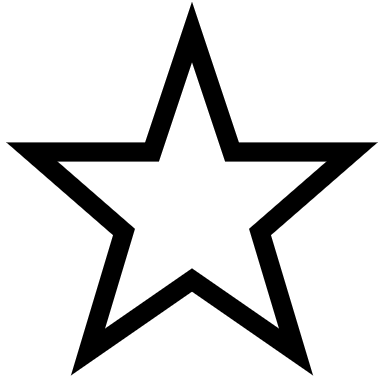

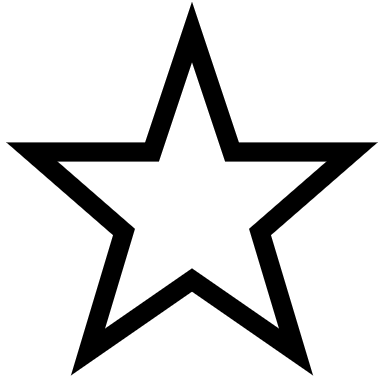

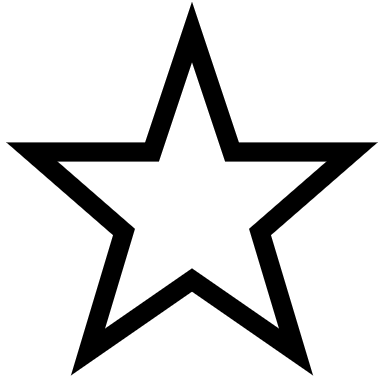

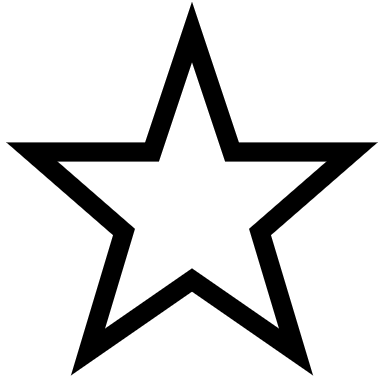

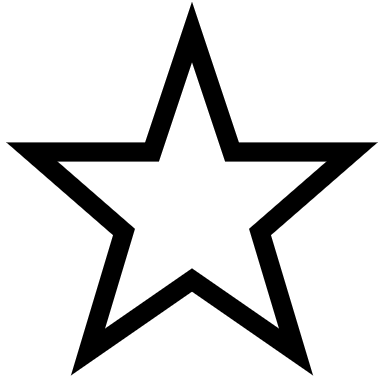
 very much*

*^#^20 If you use one or more technological devices, please tell us which one(s):*

*You may also leave free comments*

*^#^21 Are you performing or have you performed rehabilitation treatment with technological tools for this function in the clinical (inpatient) setting? **

- *No, and I'm fine with that*
- *No, but I would like to*
- *Yes, but I don't like it*
- *Yes, and I like it*

*^#^22 Are you performing or have you performed rehabilitation treatment with technological tools for this function in an outpatient clinical setting (in day hospital)?**

- *No, and I'm fine with that*
- *No, but I would like to*
- *Yes, but I don't like it*
- *Yes, and I like it*

*^#^23 Are you performing or have you performed rehabilitation treatment with technological tools for this function at home? **

- *No, and I'm fine with that*
- *No, but I would like to*
- *Yes, but I don't like it*
- *Yes, and I like it*

*24 Are you interested in learning more about it? **

- *No, I’m not working with technology and I’m not interested in knowing more*
- *Yes, even though I’m not currently working with technology, I would like to know more and will ask my referral specialist*

## Cognitive and neuropsychological domain

*In this section, we refer to cognitive and neuropsychological functions, i.e., those processes involved in the behaviours required, for example, to:*

*- find solutions to questions or situations by identifying and analysing problems;*

*- make a choice between several options and evaluate the effects of the choice, (e.g., selecting and purchasing a specific item, or deciding to undertake one activity among several ones to be performed);*

*- perform simple or complex coordinated actions to plan, manage and complete daily tasks, such as time planning and scheduling different activities during the day.*

*25 What is your level of autonomy for cognitive tasks? **

- *I totally need assistance, help and facilitation from someone else*
- *I need several tasks simplified and someone to help me*
- *I am independent and have a certain degree of autonomy, in various situations I need help*
- *I am independent and have a good degree of autonomy, I ask for a little help only in selected situations*
- *I am totally independent and have an excellent degree of autonomy*

*26 What impact does your level of autonomy in cognitive tasks have on your life?**

- *I think it's a big problem*
- *It prevents me from doing certain things and I feel sorry about it*
- *Although I can count on the help of people or the simplification of certain tasks, to me it is a big problem*
- *It is a small problem because I have functional solutions (e.g., I have someone helping me or tasks are adapted to my functioning)*
- *I don't think it is a problem at all*

*27 Are you working or have you worked in the last year on this function with a traditional rehabilitation treatment (i.e., without using technological solutions)? **

- *Not at all and that's OK*
- *Not at all but I would like to work on it*
- *Partially (e.g., no more than once a week)^#^*
- *Fairly (2 or more times a week) ^#^*
- *Very much (daily) ^#^*

*^#^28 How satisfied are/were you with this type of treatment? **

*not at all
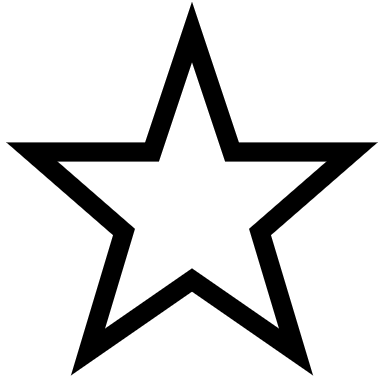

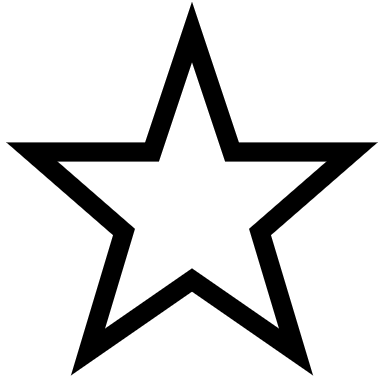

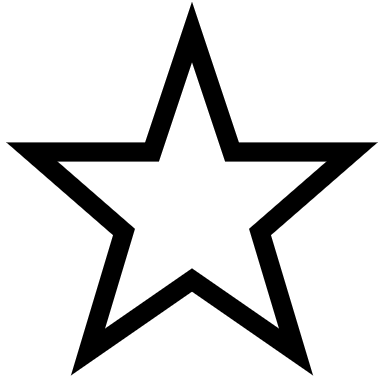

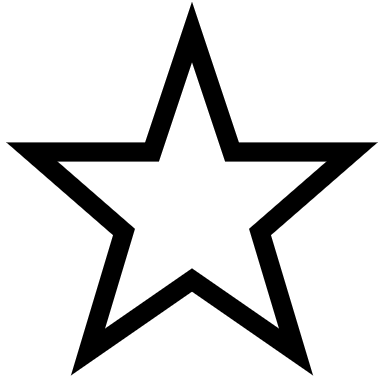

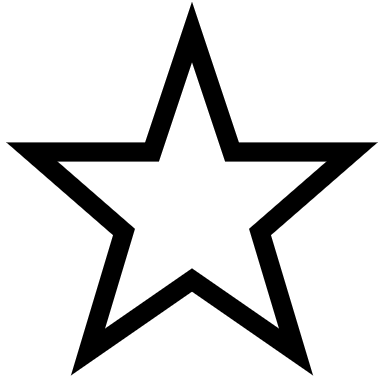
 very much*

*^#^29 Are you performing or have you performed a traditional rehabilitation treatment for this function within a clinical (inpatient) setting?*

- *No, and I'm fine with that*
- *No, but I would like to*
- *Yes, but I don't like it*
- *Yes, and I like it*

*^#^30 Are you performing or have you performed a traditional rehabilitation treatment for this function in an outpatient clinical setting (in day hospital)?**

- *No, and I'm fine with that*
- *No, but I would like to*
- *Yes, but I don't like it*
- *Yes, and I like it*

*^#^31 Are you performing or have you performed a traditional rehabilitation treatment for this function at home? **

- *No, and I'm fine with that*
- *No, but I would like to*
- *Yes, but I don't like it*
- *Yes, and I like it*

*32 Are you working or have you worked in the last year on this function with a rehabilitation/assistive treatment involving technology?*

*(an example could be a tablet with virtual reality for simulating certain tasks such as memory or attention activities)*

- *Yes^#^*
- *No*

*^#^33 How much are you working or have you worked in the last year on this function with a rehabilitation/assistive treatment involving the use of technology? **

- *Partially (e.g., no more than once a week)*
- *Fairly (2 or more times a week)*
- *A lot (every day)*

*^#^34 How satisfied are/were you with this type of treatment? **

*not at all
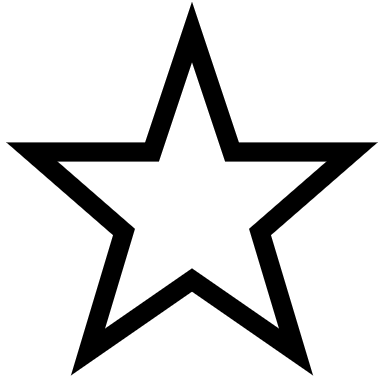

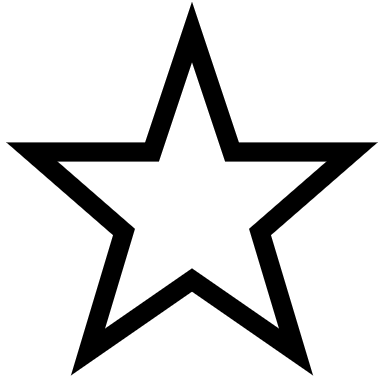

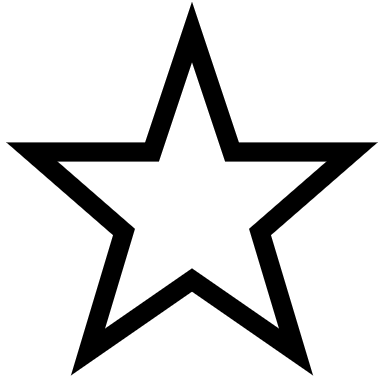

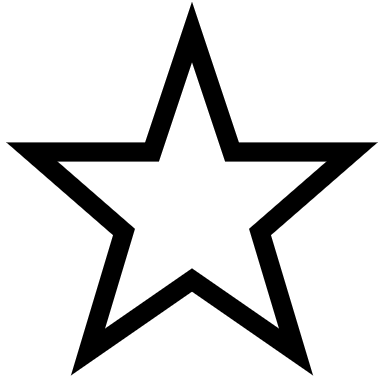

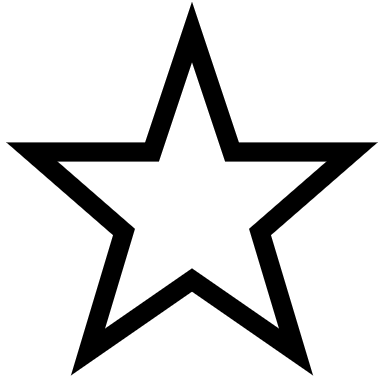
 very much*

*^#^35 If you use one or more technological devices, please tell us which one(s):*

*You may also leave free comments*

*^#^36 Are you performing or have you performed rehabilitation treatment with technological tools for this function in the clinical (inpatient) setting? **

- *No, and I'm fine with that*
- *No, but I would like to*
- *Yes, but I don't like it*
- *Yes, and I like it*

*^#^37 Are you performing or have you performed rehabilitation treatment with technological tools for this function in an outpatient clinical setting (in day hospital)?**

- *No, and I'm fine with that*
- *No, but I would like to*
- *Yes, but I don't like it*
- *Yes, and I like it*

*^#^38 Are you performing or have you performed rehabilitation treatment with technological tools for this function at home? **

- *No, and I'm fine with that*
- *No, but I would like to*
- *Yes, but I don't like it*
- *Yes, and I like it*

*39 Are you interested in learning more about it? **

- *No, I’m not working with technology and I’m not interested in knowing more*
- *Yes, even though I’m not currently working with technology, I would like to know more and will ask my referral specialist*

## Communication domain

*This section refers to functions related to verbal and non-verbal communication such as:*

*- understanding the literal and implicit meanings of messages in spoken language, such as whether a statement refers to a fact or is an idiomatic expression;*

*- producing words, and sentences with different lengths to express a fact or tell a story orally;*

*- starting, sustaining and ending a conversation; conversing with one or more persons*

*- using devices, techniques and other means to communicate, such as calling a friend on the phone;*

*- using telecommunication devices, technology for writing or speaking*

*- using gestures, symbols and drawings to convey messages, such as shaking one's head to indicate disagreement or drawing a picture to convey a complex fact or idea, but also produce or use gestures, signs, symbols, drawings and photographs*

*40 What is your level of autonomy for communication? **

- *I am dependent on others or do not communicate or rarely do so only with family members*
- *I independently manage communication devices (e.g., eye pointer, communication tables, etc.)*
- *I communicate with familiar people and most often in familiar contexts, rarely with those I do not know, I can use devices for communication*
- *I communicate autonomously, understand messages and can interact with people in various contexts even if sometimes with slowness or some uncertainty*
- *I communicate independently, understand messages and can interact with different people in different contexts*

*41 What impact does the way you communicate with others have in your life? **

- *I think it is a big problem*
- *It prevents me from doing some things and I am sorry*
- *Although I can rely on some aids (e.g., communication tables) it is a big problem for me*
- *It is a small problem because I have functional solutions (e.g., effective use of compensatory devices)*
- *I don't think it is a problem at all*

*42 Are you working or have you worked in the last year on this function with a traditional rehabilitation treatment (i.e., without using technological solutions)? **

- *Not at all and that's OK*
- *Not at all but I would like to work on it*
- *Partially (e.g., no more than once a week) ^#^*
- *Fairly (2 or more times a week) ^#^*
- *A lot (every day) ^#^*

*^#^43 How satisfied are/were you with this type of treatment? **

*not at all
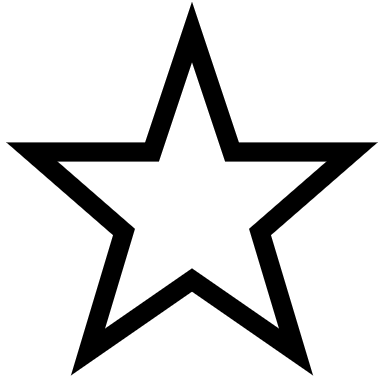

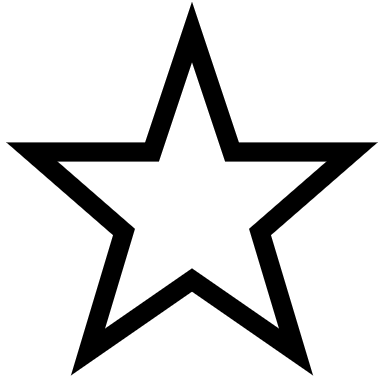

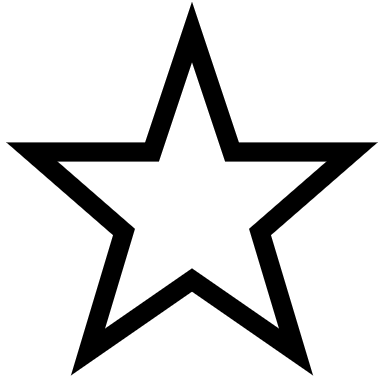

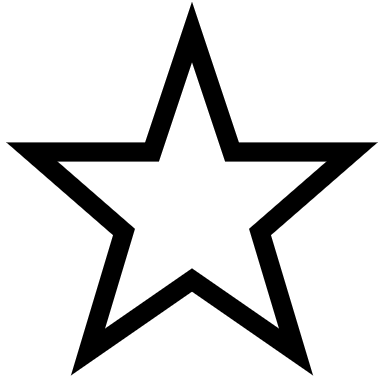

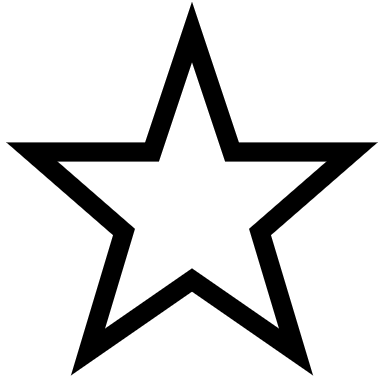
 very much*

*^#^44 Are you performing or have you performed a traditional rehabilitation treatment for this function within a clinical (inpatient) setting?*

- *No, and I'm fine with that*
- *No, but I would like to*
- *Yes, but I don't like it*
- *Yes, and I like it*

*^#^45 Are you performing or have you performed a traditional rehabilitation treatment for this function in an outpatient clinical setting (in day hospital)?**

- *No, and I'm fine with that*
- *No, but I would like to*
- *Yes, but I don't like it*
- *Yes, and I like it*

*^#^46 Are you performing or have you performed a traditional rehabilitation treatment for this function at home? **

- *No, and I'm fine with that*
- *No, but I would like to*
- *Yes, but I don't like it*
- *Yes, and I like it*

*47 Are you working or have you worked in the last year on this function with a rehabilitation/assistive treatment involving technology?*

*(an example could be a tablet with an integrated alternative augmentative communication program) **

- *Yes^#^*
- *No*

*^#^48 How much are you working or have you worked in the last year on this function with a rehabilitation/assistive treatment involving the use of technology? **

- *Partially (e.g., no more than once a week)*
- *Fairly (2 or more times a week)*
- *A lot (every day)*

*^#^49 How satisfied are/were you with this type of treatment? **

*not at all
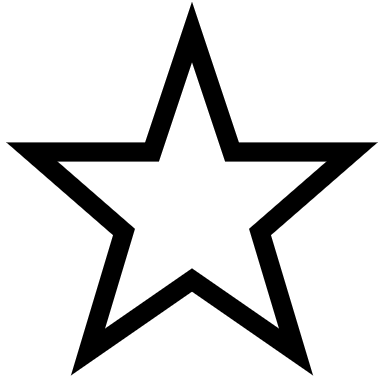

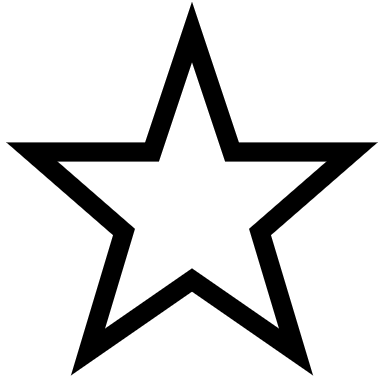

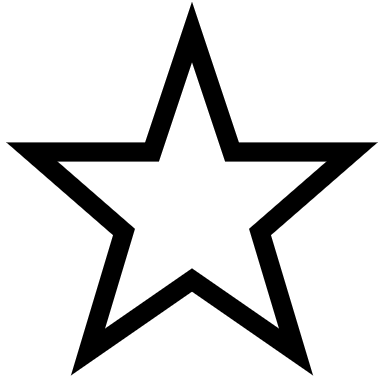

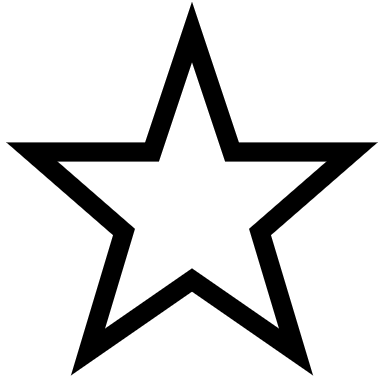

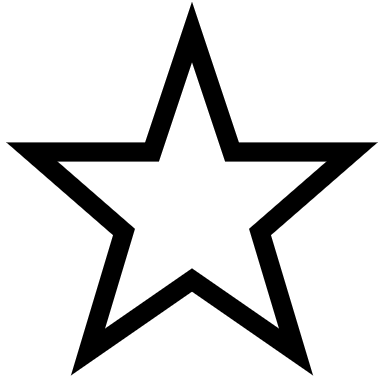
 very much*

*^#^50 If you use one or more technological devices, please tell us which one(s):*

*You may also leave free comments*

*^#^51 Are you performing or have you performed rehabilitation treatment with technological tools for this function in the clinical (inpatient) setting? **

- *No, and I'm fine with that*
- *No, but I would like to*
- *Yes, but I don't like it*
- *Yes, and I like it*

*^#^52 Are you performing or have you performed rehabilitation treatment with technological tools for this function in an outpatient clinical setting (in day hospital)?**

- *No, and I'm fine with that*
- *No, but I would like to*
- *Yes, but I don't like it*
- *Yes, and I like it*

*^#^53 Are you performing or have you performed rehabilitation treatment with technological tools for this function at home? **

- *No, and I'm fine with that*
- *No, but I would like to*
- *Yes, but I don't like it*
- *Yes, and I like it*

*54 Are you interested in learning more about it? **

- *No, I’m not working with technology and I’m not interested in knowing more*
- *Yes, even though I’m not currently working with technology, I would like to know more and will ask my referral specialist*

## Posture domain

*This section refers to the functions involved in assuming, maintaining and possibly changing different postures, e.g., supine, on one's side, sitting, standing, kneeling, etc.*

*55 What is your level of autonomy concerning body posture?**

- *I constantly need the help of aids or people to maintain a sitting posture as well*
- *I have a posture aid on which I am dependent (e.g., wheelchair), sometimes I can stand up with support*
- *I often need support, e.g., a handrest if I am standing or a backrest if I am sitting*
- *I can maintain all postures but can sometimes get fatigued or need minimal support*
- *I can maintain my posture in all situations (e.g., on one foot, sitting without a backrest).*

*56 What impact does the way you manage your posture have on your life? **

- *I think it's a big problem*
- *It prevents me from doing certain things and I feel sad about it*
- *Although I can rely on some aids (e.g. wheelchair or canes) it is a big problem for me*
- *It is a small problem because it only affects a few situations in my life and I have solutions to remedy the problem*
- *I think it is not a problem at all*

*57 Are you working or have you worked in the last year on this function with a traditional rehabilitation treatment (i.e., without using technological solutions)? **

- *Not at all and that's OK*
- *Not at all but would like to work on it*
- *Partially (e.g. no more than once a week) ^#^*
- *Fairly (2 or more times a week) ^#^*
- *Very much (daily) ^#^*

*^#^58 How satisfied are/were you with this type of treatment?**

*not at all
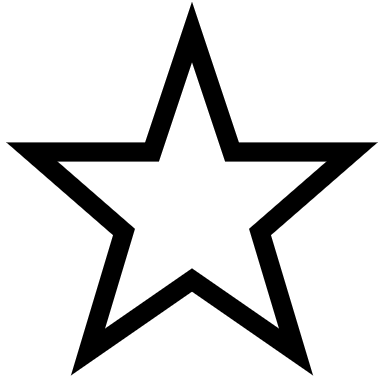

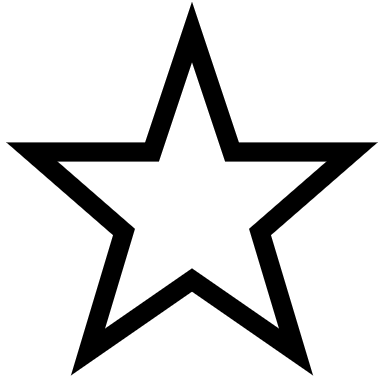

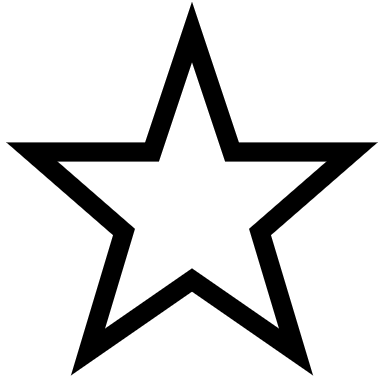

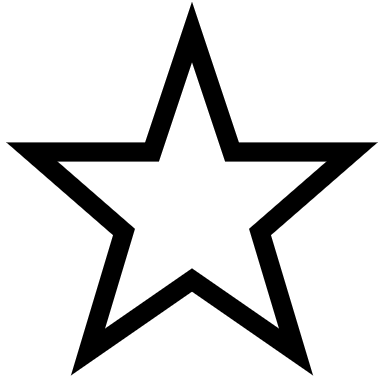

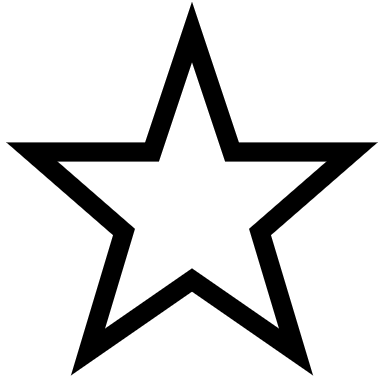
 very much*

*^#^59 Are you performing or have you performed a traditional rehabilitation treatment for this function within a clinical (inpatient) setting?*

- *No, and I'm fine with that*
- *No, but I would like to*
- *Yes, but I don't like it*
- *Yes, and I like it*

*^#^60 Are you performing or have you performed a traditional rehabilitation treatment for this function in an outpatient clinical setting (in day hospital)?**

- *No, and I'm fine with that*
- *No, but I would like to*
- *Yes, but I don't like it*
- *Yes, and I like it*

*^#^61 Are you performing or have you performed a traditional rehabilitation treatment for this function at home? **

- *No, and I'm fine with that*
- *No, but I would like to*
- *Yes, but I don't like it*
- *Yes, and I like it*

*62 Are you working or have you worked in the last year on this function with a rehabilitation/assistive treatment involving technology?*

*(an example could be a device to assist with postural changes, such as a lift.) **

- *Yes^#^*
- *No*

*^#^63 How much are you working or have you worked in the last year on this function with a rehabilitation/assistive treatment involving the use of technology? **

- *Partially (e.g., no more than once a week)*
- *Fairly (2 or more times a week)*
- *A lot (every day)*

*^#^64 How satisfied are/were you with this type of treatment?**

*not at all
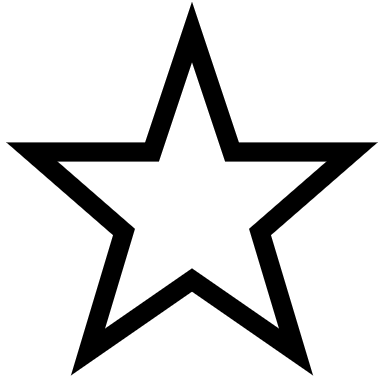

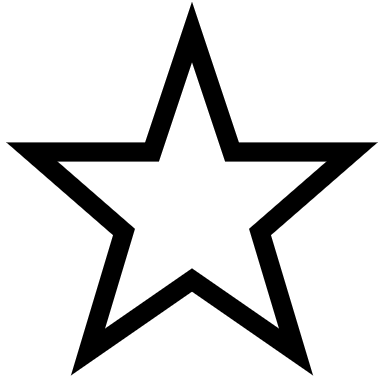

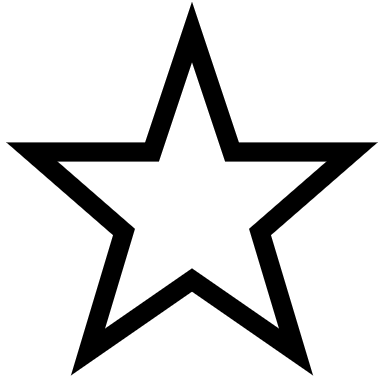

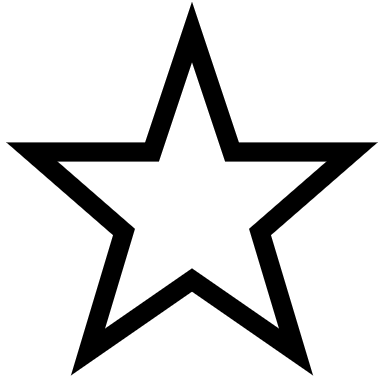

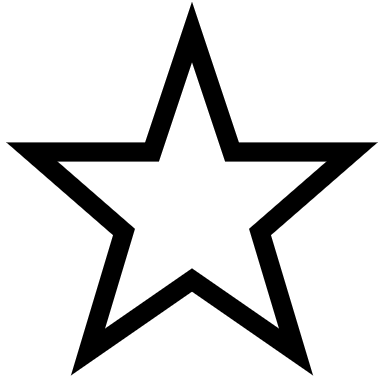
 very much*

*^#^65 If you use one or more technological devices, please tell us which one(s):*

*You may also leave free comments*

*^#^66 Are you performing or have you performed rehabilitation treatment with technological tools for this function in the clinical (inpatient) setting? **

- *No, and I'm fine with that*
- *No, but I would like to*
- *Yes, but I don't like it*
- *Yes, and I like it*

*^#^67 Are you performing or have you performed rehabilitation treatment with technological tools for this function in an outpatient clinical setting (in day hospital)?**

- *No, and I'm fine with that*
- *No, but I would like to*
- *Yes, but I don't like it*
- *Yes, and I like it*

*^#^68 Are you performing or have you performed rehabilitation treatment with technological tools for this function at home? **

- *No, and I'm fine with that*
- *No, but I would like to*
- *Yes, but I don't like it*
- *Yes, and I like it*

*69 Are you interested in learning more about it? **

- *No, I’m not working with technology and I’m not interested in knowing more*
- *Yes, even though I’m not currently working with technology, I would like to know more and will ask my referral specialist*

## Self-care domain

*This section refers to the functions involved in performing tasks related to daily routines such as preparing food and eating, pouring drinks or mixing and drinking, dressing, washing, and taking care of your body.*

*70 What is your level of autonomy for personal care (e.g. eating, drinking, washing, body care, dressing)?**

- *I am completely dependent on someone else*
- *I need help and/or solutions that help me (e.g., handles in the bathroom, facilitated clothing, adapted cutlery, etc.).*
- *I can do some things independently but more often I use aids (e.g., handles in the bathroom, facilitated clothing, adapted cutlery).*
- *I have good autonomy but can sometimes benefit from some adjustments in the material I use*
- *I am completely independent*

*71 What impact does the way you take care of yourself have on your life? **

- *It's a big problem*
- *It prevents me from doing some things and I feel sad*
- *Although I can count on some adaptations (e.g., handles or adapted material) it is a big problem for me*
- *It's a small problem because I have functional solutions*
- *I don't think it is a problem at all*

*72 Are you working or have you worked in the last year on this function with a traditional rehabilitation treatment (i.e., without using technological solutions)? **

- *Not at all and that's OK*
- *Not at all, but would like to work on it*
- *Partially (e.g., no more than once a week) ^#^*
- *Fairly (2 or more times a week) ^#^*
- *Very much (daily) ^#^*

*^#^73 How satisfied are/were you with this type of treatment?**

*not at all
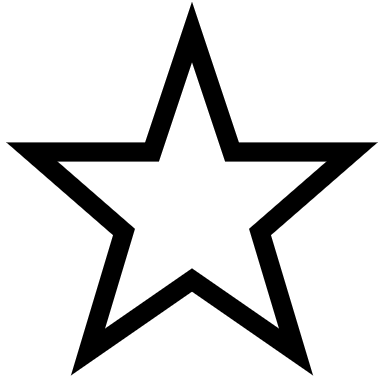

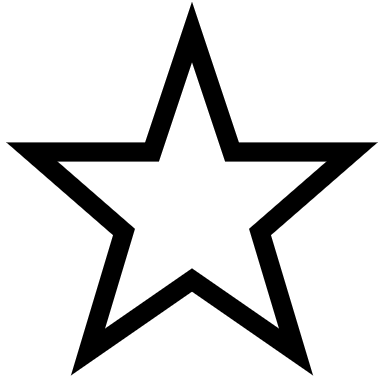

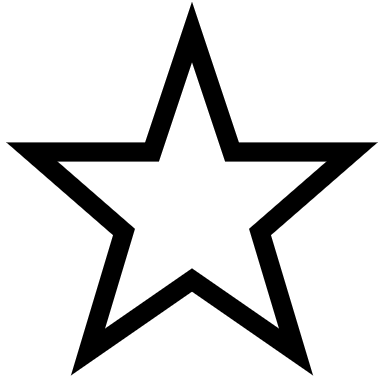

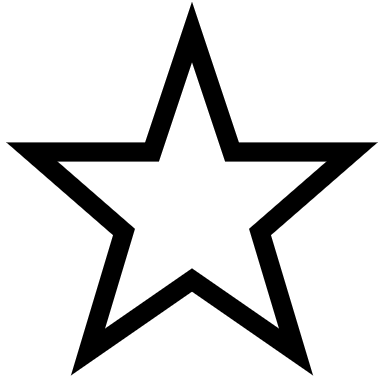

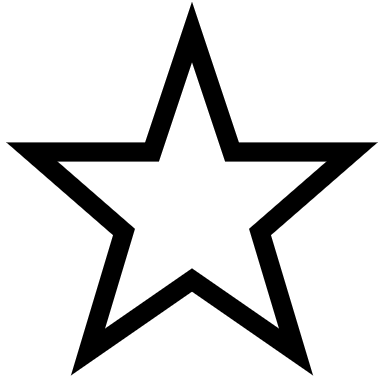
 very much*

*^#^74 Are you performing or have you performed a traditional rehabilitation treatment for this function within a clinical (inpatient) setting?*

- *No, and I'm fine with that*
- *No, but I would like to*
- *Yes, but I don't like it*
- *Yes, and I like it*

*^#^75 Are you performing or have you performed a traditional rehabilitation treatment for this function in an outpatient clinical setting (in day hospital)?**

- *No, and I'm fine with that*
- *No, but I would like to*
- *Yes, but I don't like it*
- *Yes, and I like it*

*^#^76 Are you performing or have you performed a traditional rehabilitation treatment for this function at home? **

- *No, and I'm fine with that*
- *No, but I would like to*
- *Yes, but I don't like it*
- *Yes, and I like it*

*77 Are you working or have you worked in the last year on this function with a rehabilitation/assistive treatment involving technology?*

*(an example could be a device that facilitates mealtimes) **

- *Yes^#^*
- *No*

*^#^78 How much are you working or have you worked in the last year on this function with a rehabilitation/assistive treatment involving the use of technology? **

- *Partially (e.g. no more than once a week)*
- *Fairly (2 or more times a week)*
- *A lot (every day)*

*^#^79 How satisfied are/were you with this type of treatment? **

*not at all
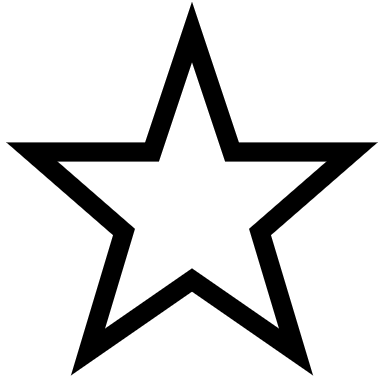

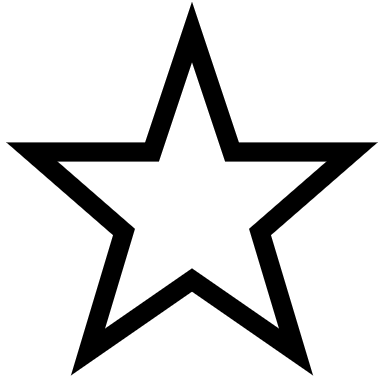

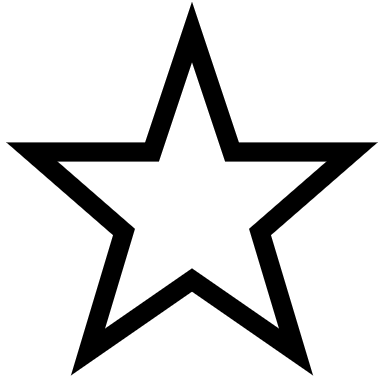

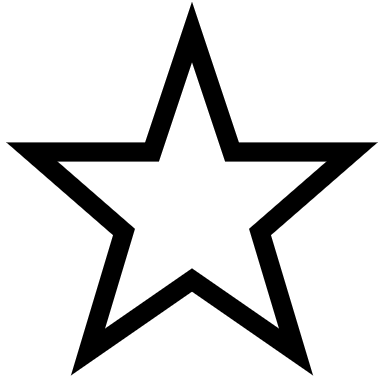

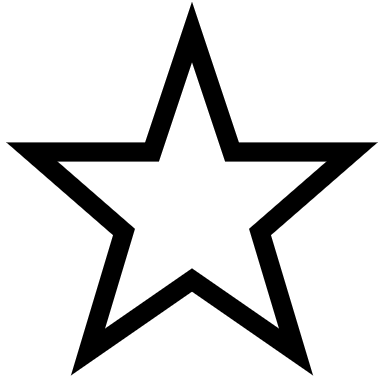
 very much*

*^#^80 If you use one or more technological devices, please tell us which one(s):*

*You may also leave free comments*

*^#^81 Are you performing or have you performed rehabilitation treatment with technological tools for this function in the clinical (inpatient) setting? **

- *No, and I'm fine with that*
- *No, but I would like to*
- *Yes, but I don't like it*
- *Yes, and I like it*

*^#^82 Are you performing or have you performed rehabilitation treatment with technological tools for this function in an outpatient clinical setting (in day hospital)?**

- *No, and I'm fine with that*
- *No, but I would like to*
- *Yes, but I don't like it*
- *Yes, and I like it*

*^#^83 Are you performing or have you performed rehabilitation treatment with technological tools for this function at home? **

- *No, and I'm fine with that*
- *No, but I would like to*
- *Yes, but I don't like it*
- *Yes, and I like it*

*84 Are you interested in learning more about it? **

- *No, I’m not working with technology and I’m not interested in knowing more*
- *Yes, even though I’m not currently working with technology, I would like to know more and will ask my referral specialist*

## Upper limb and hands use domain

*This section refers to functions related to the use of the hands and arms including:*

*- performing coordinated actions to handle and manipulate objects, such as lifting coins or turning knobs using your hands, fingers, and thumbs.*

*- performing coordinated actions required to move objects or manipulate them using hands and arms, e.g., when turning door handles or throwing or grasping an object, pulling or pushing objects*

*85 What is your level of independence in the use of your upper limbs and hands?**

- *I need total assistance even for simple actions*
- *I can only manipulate certain objects in selected situations and/or that are adapted to me (facilitated)*
- *I handle many objects independently but can benefit from material adaptations and/or help*
- *I handle many different objects even though slowly or with uncertainty and sometimes incomplete precision*
- *I am independent in most handling activities*

*86 What impact does the way you use your arms and hands have in your life? **

- *It's a big problem*
- *It prevents me from doing some activities and I feel sad*
- *Although I can rely on the adaptation of materials or help from others, it is a big problem for me*
- *It is a small problem because I have functional solutions (e.g. I use facilitated objects or despite being slow I am successful in actions)*
- *I don't think it is a problem at all*

*87 Are you working or have you worked in the last year on this function with a traditional rehabilitation treatment (i.e. without using technological solutions)? **

- *Not at all and that's OK*
- *Not at all but would like to work on it*
- *Partially (e.g. no more than once a week) ^#^*
- *Fairly (2 or more times a week) ^#^*
- *Very much (daily) ^#^*

*^#^88 How satisfied are/were you with this type of treatment?**

*not at all
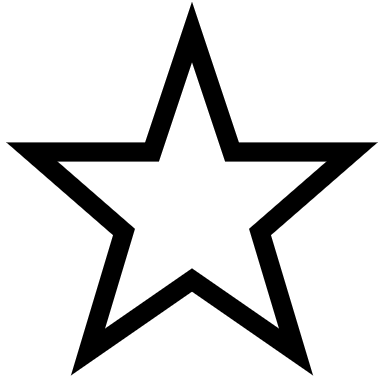

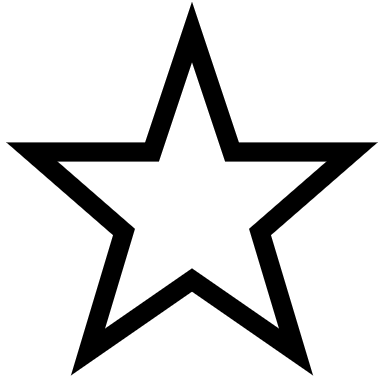

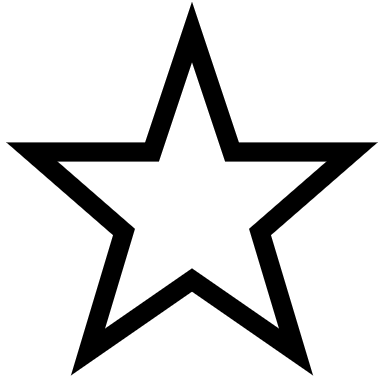

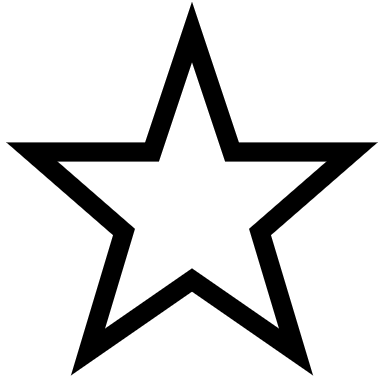

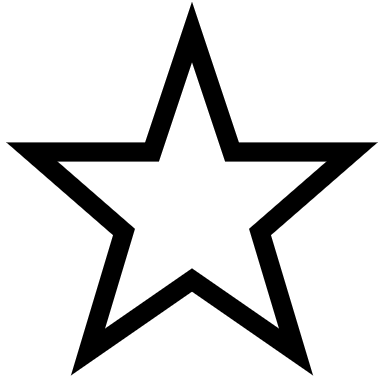
 very much*

*^#^89 Are you performing or have you performed a traditional rehabilitation treatment for this function within a clinical (inpatient) setting?*

- *No, and I'm fine with that*
- *No, but I would like to*
- *Yes, but I don't like it*
- *Yes, and I like it*

*^#^90 Are you performing or have you performed a traditional rehabilitation treatment for this function in an outpatient clinical setting (in day hospital)?**

- *No, and I'm fine with that*
- *No, but I would like to*
- *Yes, but I don't like it*
- *Yes, and I like it*

*^#^91 Are you performing or have you performed a traditional rehabilitation treatment for this function at home? **

- *No, and I'm fine with that*
- *No, but I would like to*
- *Yes, but I don't like it*
- *Yes, and I like it*

*92 Are you working or have you worked in the last year on this function with a rehabilitation/assistive treatment involving technology?*

*(an example could be the use of an exoskeleton for the upper limb) **

- *Yes^#^*
- *No*

*^#^93 How much are you working or have you worked in the last year on this function with a rehabilitation/assistive treatment involving the use of technology? **

- *Partially (e.g. no more than once a week)*
- *Fairly (2 or more times a week)*
- *A lot (every day)*

*^#^94 How satisfied are/were you with this type of treatment?**

*not at all
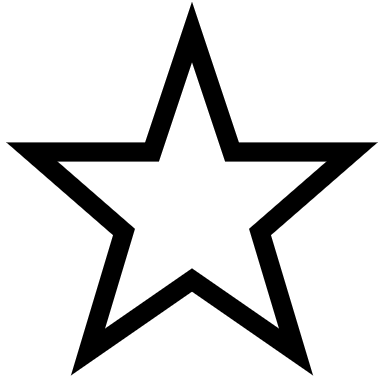

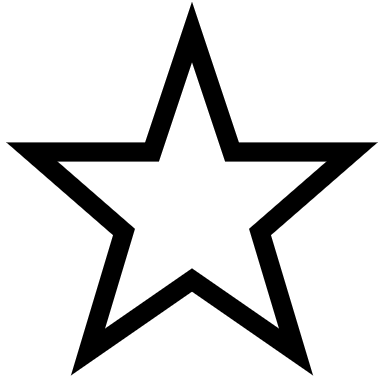

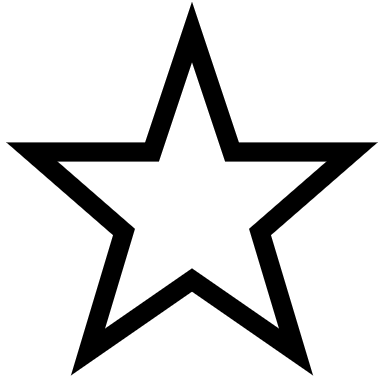

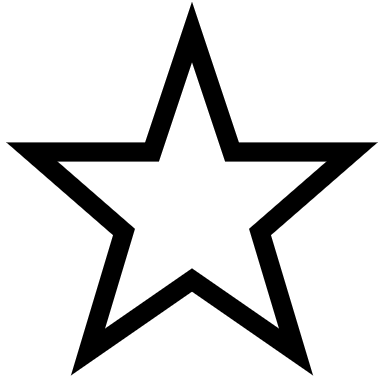

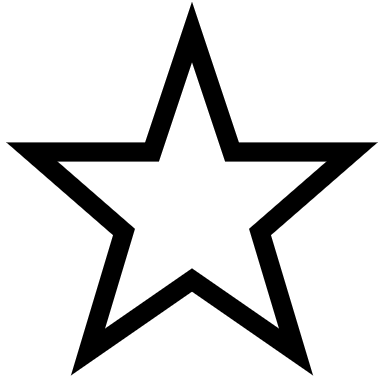
 very much*

*^#^95 If you use one or more technological devices, please tell us which one(s):*

*You may also leave free comments*

*^#^96 Are you performing or have you performed rehabilitation treatment with technological tools for this function in the clinical (inpatient) setting? **

- *No, and I'm fine with that*
- *No, but I would like to*
- *Yes, but I don't like it*
- *Yes, and I like it*

*^#^97 Are you performing or have you performed rehabilitation treatment with technological tools for this function in an outpatient clinical setting (in day hospital)?**

- *No, and I'm fine with that*
- *No, but I would like to*
- *Yes, but I don't like it*
- *Yes, and I like it*

*^#^98 Are you performing or have you performed rehabilitation treatment with technological tools for this function at home? **

- *No, and I'm fine with that*
- *No, but I would like to*
- *Yes, but I don't like it*
- *Yes, and I like it*

*99 Are you interested in learning more about it? **

- *No, I’m not working with technology and I’m not interested in knowing more*
- *Yes, even though I’m not currently working with technology, I would like to know more and will ask my referral specialist*

## Types of treatments

*100 Are/were all the treatments dispensed by the National Health Service?*

- *Yes*
- *No*

*101 Please tell us how much you spent on average in the last year on:*

***Technologies/devices*** *(e.g., rehabilitation devices purchased or rented privately, other devices such as a blood pressure monitor, wheelchair, equipped bed, adapters for the toilet, bathtub, shower, etc.) **

- *0 €*
- *< 500 €*
- *between 501 € and 2000 €*
- *between 2001 € and 5000 €*
- *> 5000 €*

*102 Please tell us how much you spent on average in the last year on:*

***Expenses for adapting the home environment*** *to his needs (e.g., ramps, furniture adapted for wheelchairs, etc.) **

- *0 €*
- *< 500 €*
- *between 501 € and 2000 €*
- *between 2001 € and 5000 €*
- *> 5000 €*

*103 Please tell us how much you spent on average in the last year on:*

***Drugs, supplements****, or generally pharmaceutical products or* ***special foods*** *not dispensed by the national health service **

- *0 €*
- *< 500 €*
- *between 501 € and 2000 €*
- *between 2001 € and 5000 €*
- *> 5000 €*

*104 Please tell us how much you spent on average in the last year on:*

***Transport/travel*** *(expenses for travel, petrol and parking, possible board and lodging, etc.). Also, consider the expenses of any family members or other unpaid caregivers assisting you in your rehabilitation **

- *0 €*
- *< 500 €*
- *between 501 € and 2000 €*
- *between 2001 € and 5000 €*
- *> 5000 €*

*105 Please tell us how much you spent on average in the last year on:*

***Assistance from*** ***non-medical personnel*** *(e.g., carer/babysitter for minors), which is necessary to pay to be able to carry out rehabilitation **

- *0 €*
- *< 500 €*
- *between 501 € and 2000 €*
- *between 2001 € and 5000 €*
- *> 5000 €*

*106 Please tell us how much you spent on average in the last year on:*

***Health care interventions for rehabilitation*** *(e.g., specialist medical examinations, physiotherapist, speech therapist, professional nurses, etc.) paid privately **

- *0 €*
- *< 500 €*
- *between 501 € and 2000 €*
- *between 2001 € and 5000 €*
- *> 5000 €*

*107 Please tell us how much you spent on average in the last year on:*

***Loss of income*** *due to lost/reduced work/no career advancement, etc.*

*Consider also your family member or other unpaid caregiver assisting you in your rehabilitation *.*

- *0 €*
- *< 500 €*
- *between 501 € and 2000 €*
- *between 2001 € and 5000 €*
- *> 5000 €*

*108 Please tell us how much you spent on average in the last year for* ***other things*** *(please specify later) **

- *0 €*
- *< 500 €*
- *between 501 € and 2000 €*
- *between 2001 € and 5000 €*
- *> 5000 €*

*109 If you selected something else, please specify the cost item you referred to in the previous question*

*110 In conclusion, how much financial hardship do you feel due to the costs of your rehabilitation?**

- *not at all*
- *less than a little*
- *a little*
- *fairly*
- *very much*

## Final question

*111 Based on what you answered for the six different areas just analysed, if you were asked to prioritise treatment, how would you order them?*

*(you can drag the boxes to put the one referring to the most important domain at the top, followed by the ones that are progressively lower in priority)*

- *use of upper limbs and hands*
- *communication*
- *cognitive/neuropsychological field*
- *movement*
- *self-care*
- *posture*

*112 Would you like to leave a free comment? **

- *No thanks, I have nothing to add*
- *Yes, gladly^#^*

*^#^113 Write down what you would like to tell us **

*Click 'send' to save your answer!*

*In this way, we could register a willingness to take part in the survey.*

*The Fit4MedRob project team*

1. www.fit4medrob.it [↑](#footnote-ref-1)
