# Supplementary material for: Identifying needs in adult rehabilitation to support the clinical implementation of robotics and allied technologies: an Italian national survey
Source: Front Digit Health. 2026 Apr 1;8:1718274. doi: 10.3389/fdgth.2026.1718274 (PMC13081780; doi:10.3389/fdgth.2026.1718274)
Supplement: Supplementary file 2 [file Datasheet2.docx]

**Identifying Needs in Adult Rehabilitation to Support the Clinical Implementation of Robotics and Allied Technologies: An Italian National Survey**

**Supplementary Material 2: *Stratified Analyses of responses***


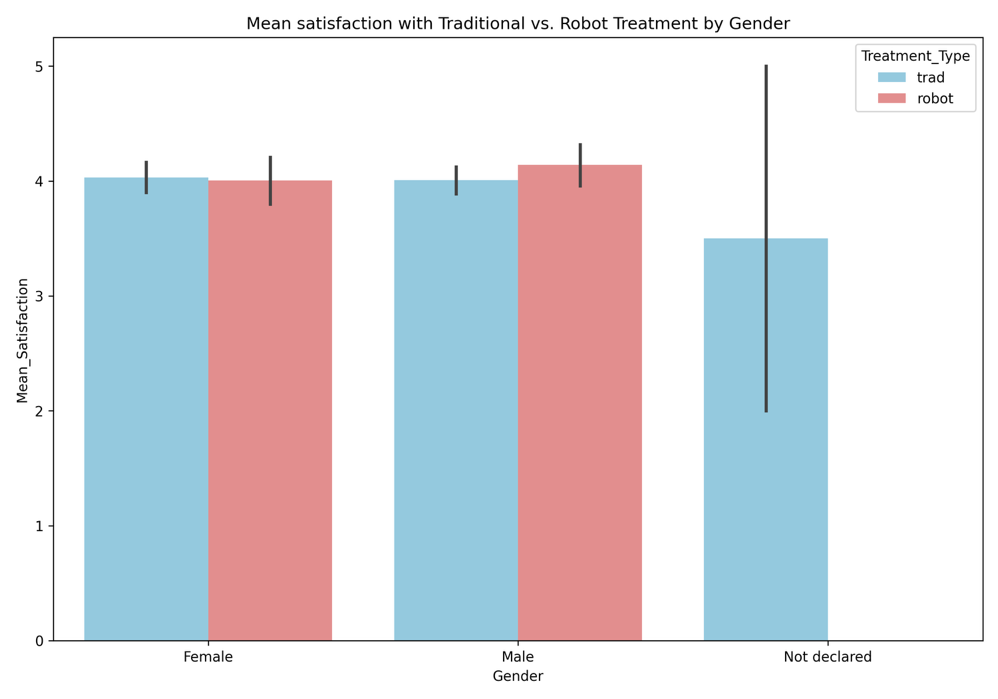


Figure S1: Mean satisfaction of different rehabilitative treatments (traditional vs robotics) stratified by gender


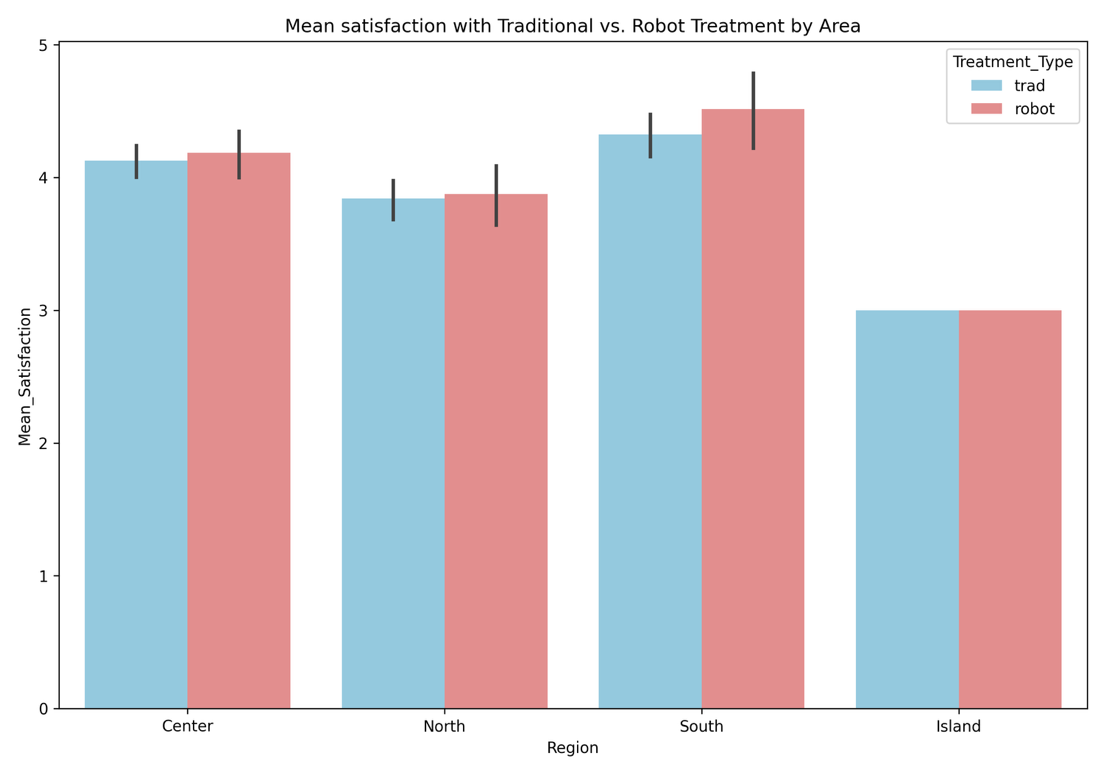


Figure S2: Mean satisfaction of different rehabilitative treatments (traditional vs robotics) stratified by geographical area


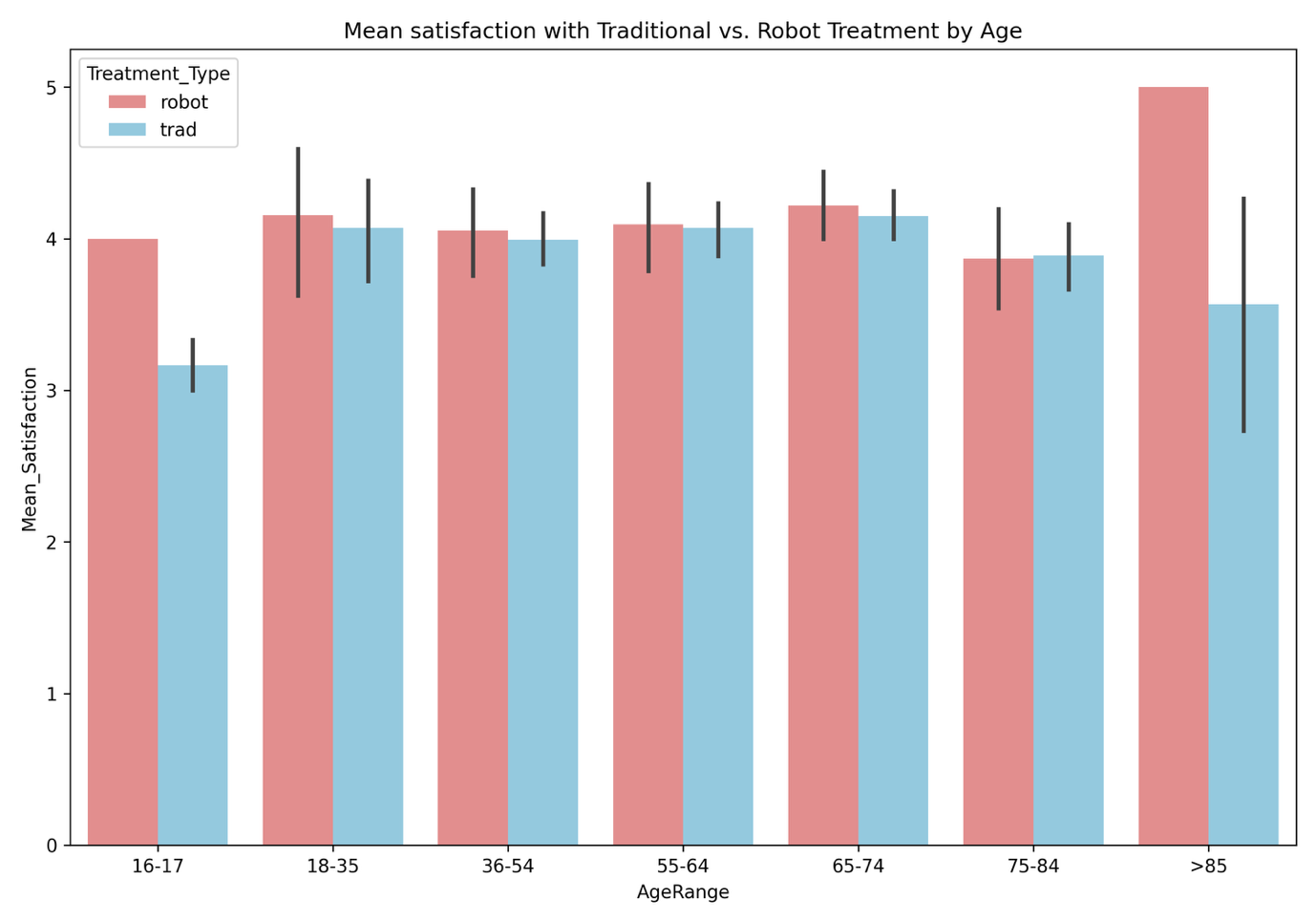


Figure S3: Mean satisfaction of different rehabilitative treatments (traditional vs robotics) stratified by age


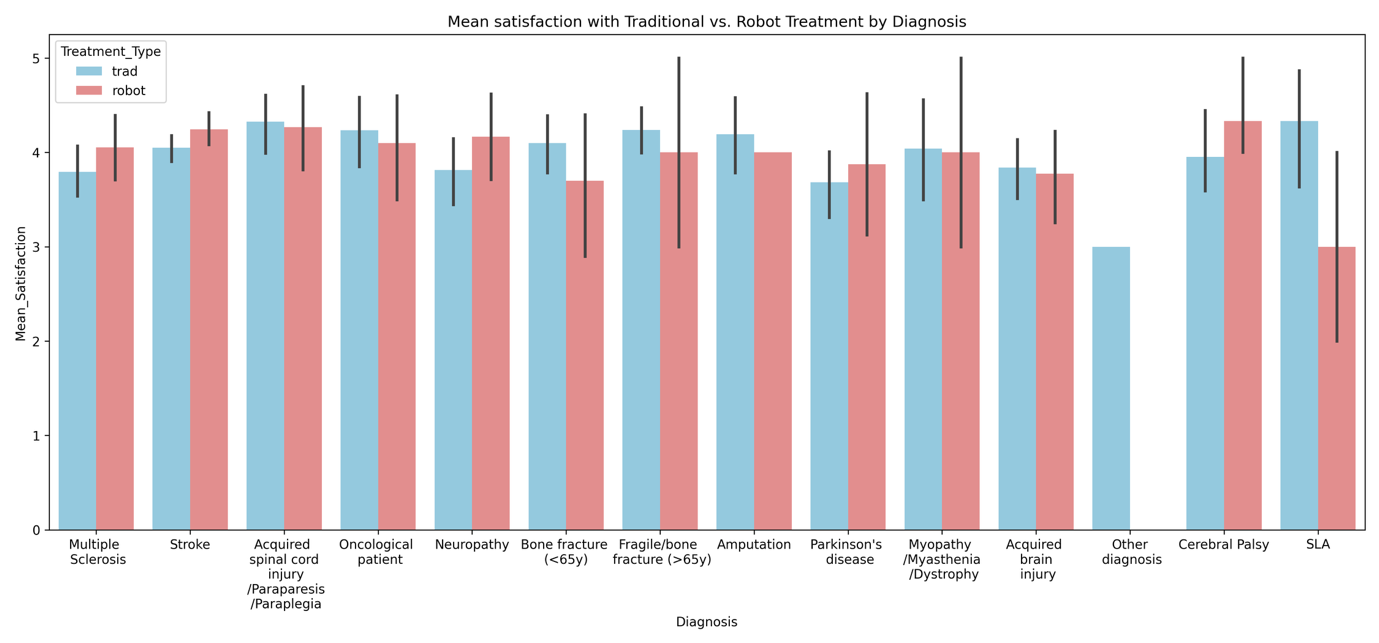


Figure S4: Mean satisfaction of different rehabilitative treatments (traditional vs robotics) stratified by diagnosis


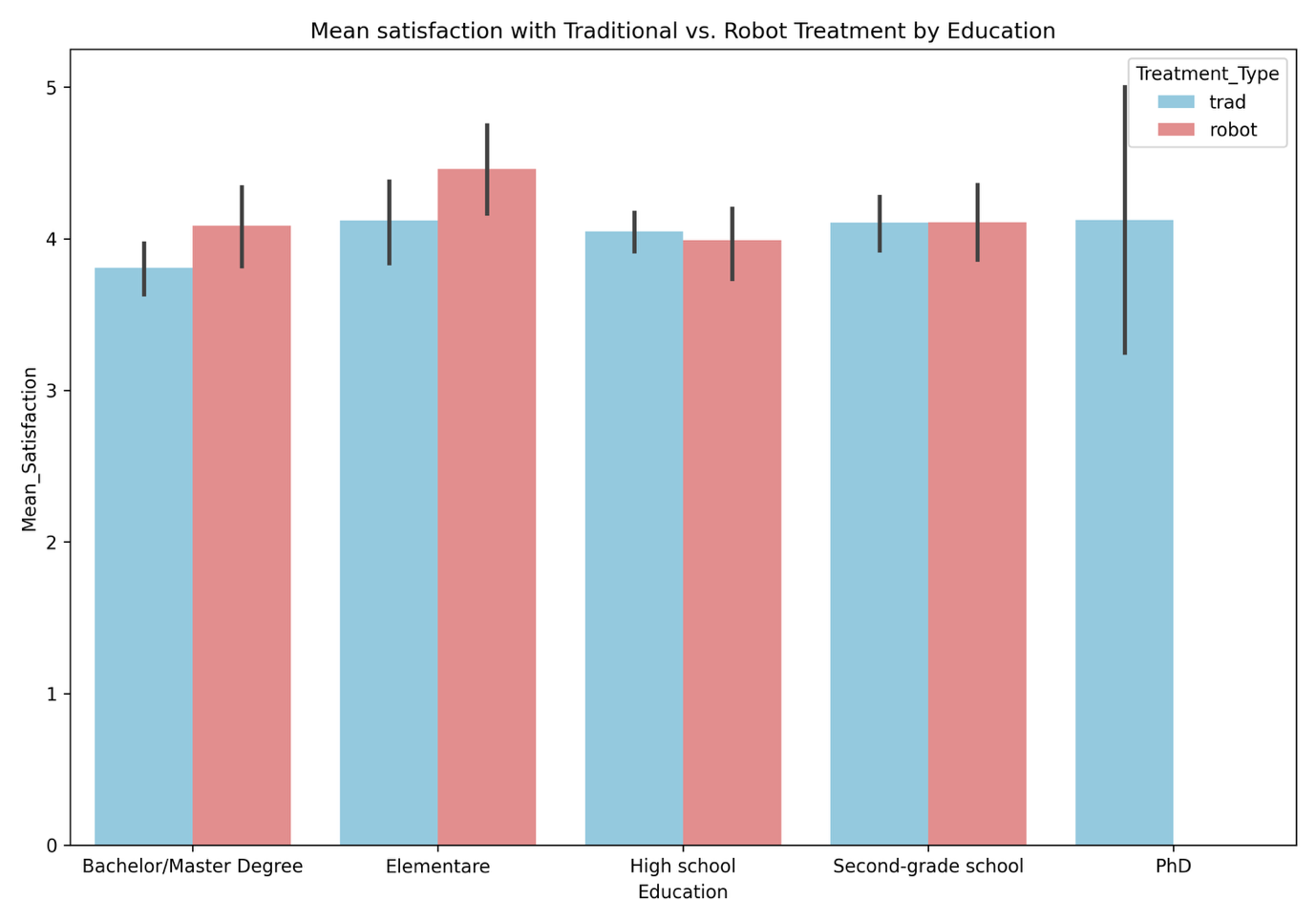


First-grade school

Figure S5: Mean satisfaction of different rehabilitative treatments (traditional vs robotics) stratified by education
